# Deciphering the SAM- and metal-dependent mechanism of O-methyltransferases in cystargolide and belactosin biosynthesis: A structure–activity relationship study

**DOI:** 10.1016/j.jbc.2024.107646

**Published:** 2024-08-08

**Authors:** Wolfgang Kuttenlochner, Patrick Beller, Leonard Kaysser, Michael Groll

**Affiliations:** 1 Department of Bioscience, Center for Protein Assemblies (CPA), TUM School of Natural Sciences, Technical University of Munich, Garching, Germany; 2 Department of Pharmaceutical Biology, Pharmaceutical Institute, University of Tübingen, Tübingen, Germany; 3 Department of Pharmaceutical Biology, Institute for Drug Discovery, University of Leipzig, Leipzig, Germany

**Keywords:** natural product biosynthesis, enzyme mechanism, metal-ion protein interaction, molecular docking, structure-function

## Abstract

Cystargolides and belactosins are natural products with a distinct dipeptide structure and an electrophilic β-lactone warhead. They are known to inhibit proteases such as the proteasome or caseinolytic protease P, highlighting their potential in treating cancers and neurodegenerative diseases. Recent genetic analyses have shown homology between the biosynthetic pathways of the two inhibitors. Here, we characterize the O-methyltransferases BelI and CysG, which catalyze the initial step of β-lactone formation. Employing techniques such as crystallography, computational analysis, mutagenesis, and activity assays, we identified a His-His-Asp (HHD) motif in the active sites of the two enzymes, which is crucial for binding a catalytically active calcium ion. Our findings thus elucidate a conserved divalent metal-dependent mechanism in both biosynthetic pathways that distinguish BelI and CysG from previously characterized O-methyltransferases.

Secondary metabolites produced by a variety of organisms, including fungi, plants, and bacteria are excellent candidates for drug development due to their unique bioactive properties (1, 2, 3, 4). Among these natural products (NPs), β-lactones (2-oxetanones) are of interest because of their antimicrobial and anticancer properties. The unique reactivity of β-lactones, attributed to their high electrophilicity and ring-strained structure, makes them suitable for targeting various enzyme classes including hydrolases, transferases, ligases, and oxidoreductases (5, 6). The proteasome core particle (CP), crucial for protein degradation, serves as a key example of such interactions. Dysregulation of CP function is attributed to several pathogenic conditions, including cancer, autoimmune diseases, and neurogenerative disorders (7, 8).

A number of β-lactone CP inhibitors exhibit a complex structural arrangement with β-lactone-γ-lactam moieties (1, 9, 10, 11). In contrast, cystargolides and belactosins have a simpler architecture consisting of a dipeptide backbone with a reactive terminal β-lactone warhead (Fig. 1*A*). Their inhibitory activity against CP involves the nucleophilic attack of the catalytic Thr1O^γ^ on the electrophilic β-lactone, resulting in the formation of a stable acyl-enzyme complex. In cystargolides and belactosins, this interaction is particularly influenced by the stereochemistry of residues at the P1 site, which results in a unique binding mode within the catalytic subunits of the proteasome (12, 13, 14). In addition, cystargolides have been shown to inhibit caseinolytic protease P (ClpP) (15), a hydrolase associated with the virulence of *Staphylococcus aureus*, *Listeria monocytogenes*, and *Pseudomonas aeruginosa* (16, 17, 18, 19, 20). On the other hand, the malfunction of ClpP in mitochondria has been implicated in cancer and neurogenerative disorders (21, 22).

**Figure 1:**
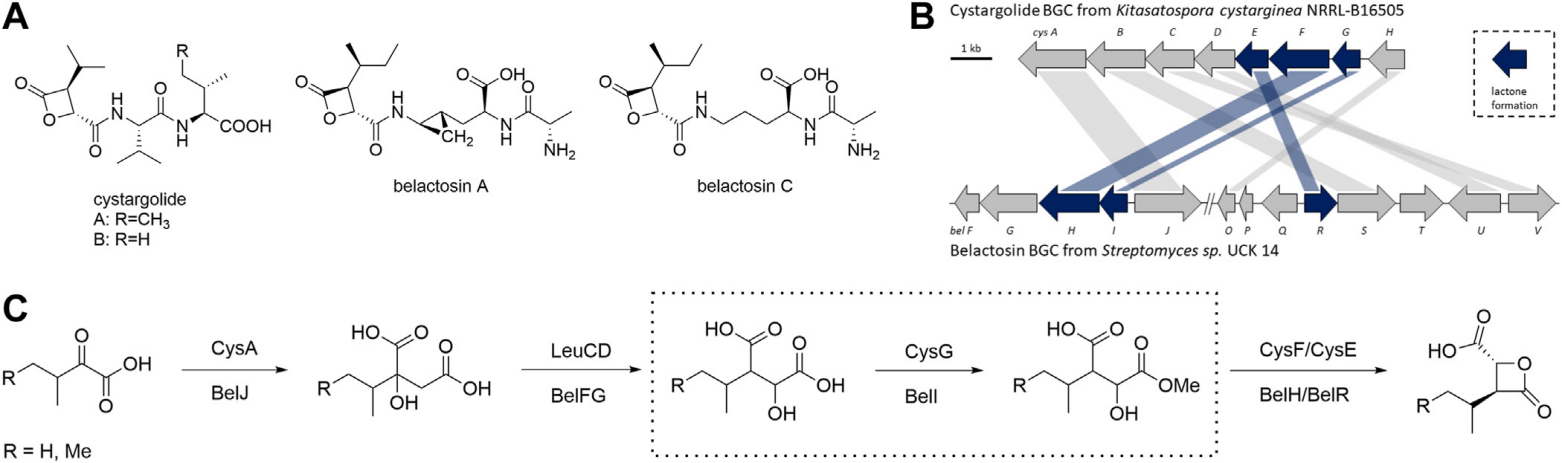
**Biosynthesis of Cystargolides and Belactosins.***A*, chemical structures of cystargolides and belactosins. *B*, gene cluster maps for cystargolide (*top*) and belactosin (*bottom*), color-coded by proposed biosynthetic functions. *C*, biosynthetic pathways for cystargolides and belactosins, highlighting their analogous routes, shared homologs with leucine biosynthesis, and the reaction of the OMTases CysG and BelI.

The majority of β-lactones are typically produced by non-ribosomal peptide synthetases (NRPSs), polyketide synthases (PKSs), or β-lactone synthetases (BLSs). While the biosynthetic gene clusters (BGCs) responsible for cystargolide and belactosin production in *Kitasatospora cystarginea* NRRL B16505 and *Streptomyces* sp. UCK 14 lack genes encoding for NRPSs or PKSs, they each contain a gene encoding for a putative BLS. However, the corresponding enzymes, CysF and BelH, exhibit only minor primary sequence identity to known BLSs and the biosynthetic pathways involve an isopropylmalate synthase homolog that parallels bacterial leucine biosynthesis (2, 12, 23, 24, 25). In addition, the *cys* and *bel* BGCs contain a *S*-adenosylmethionine (SAM)-dependent O-methyltransferase (OMTase; CysG/BelI), a methylesterase (CysE/BelR) and ATP-dependent enzymes (CysC, CysD, CysF/BelU, BelV, BelH) (Fig. 1*B*) (26).

β-lactone biosynthesis starts with methylation, likely followed by activation for lactonization *via* ATP, and concludes with cleavage of the methyl ester in a sequential reaction cascade. The final step involves coupling the β-lactone warhead with the peptide backbone catalyzed by various carboxy-amino ligases, respectively (26, 27). Our recent study characterized the methylation products of the OMTases CysG and BelI by NMR analysis (24, 27). Here, we use an integrative approach, combining crystallography, computational modeling, mutagenesis, and functional analysis to provide atomic insights into the initial catalytic process of β-lactone warhead formation (Fig. 1*C*).

## Results

**Figure 2:**
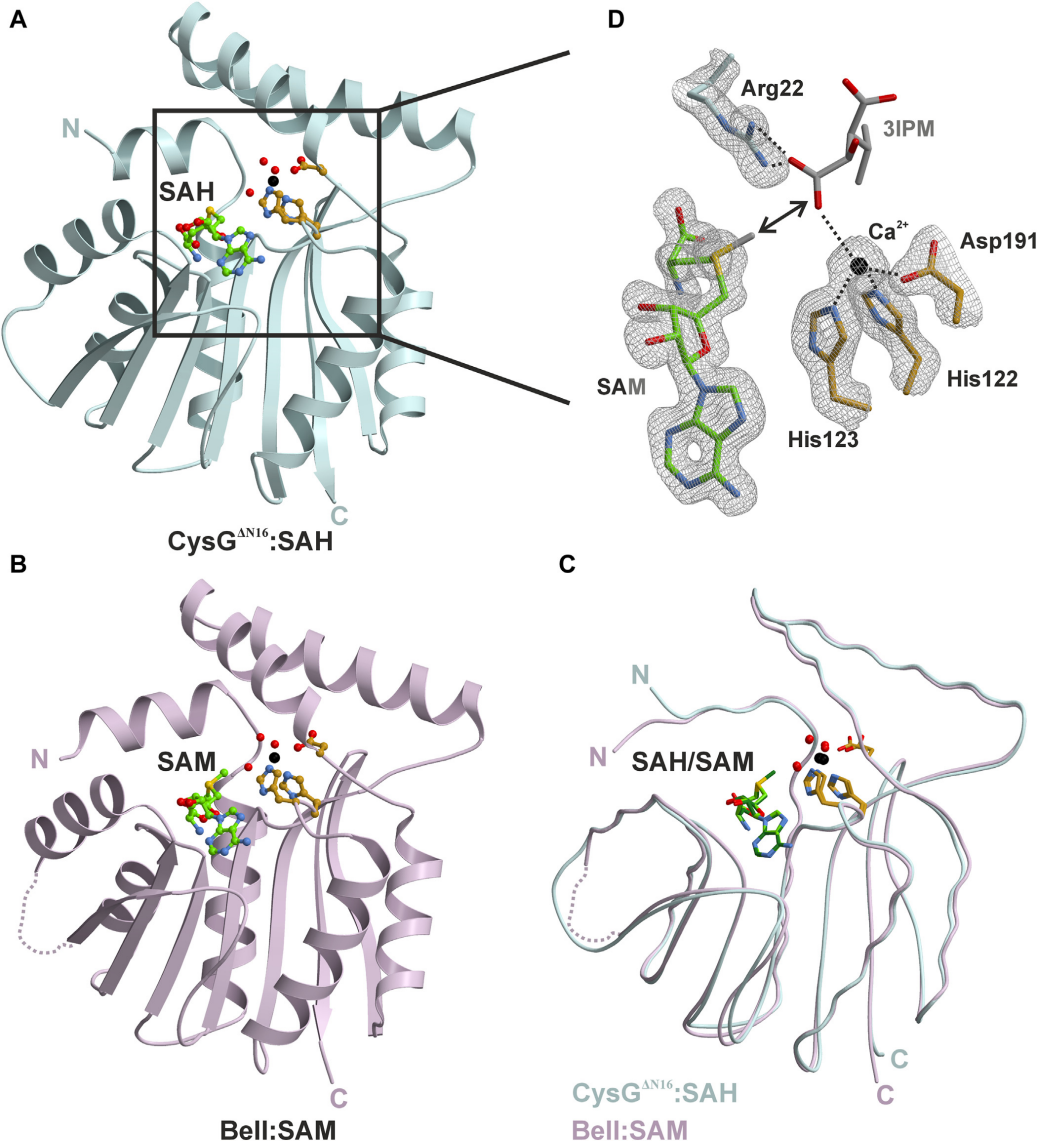
**Crystal structure of CysG**^**ΔN16**^**and BelI.***A*, cartoon representation of CysG^ΔN16^ in complex with SAH (PDB ID 9FCD, carbon atoms in *green*, oxygen in *red*, nitrogen in *blue*). Calcium is coordinated in an octahedral coordination sphere by the HHD residues and three water molecules (carbon atoms in gold, Ca^2+^ in *black*). The *rectangle* indicates the section used for molecular docking calculations. *B*, cartoon representation of BelI in complex with SAM and calcium bound by the HDD motif (PDB ID 9FCE, C^α^-trace shown in magenta, water molecules in *red*). *C*, superposition of BelI (*magenta*) and CysG^ΔN16^ (*cyan*) in complex with SAM/SAH reveal high structural homology with a rmsd of 0.6 Å. *D*, illustration of the active site of CysG^ΔN16^. The 2F_O_-F_C_ electron density map (*grey mesh*; contoured to 1σ) depicts the binding of SAH and a calcium ion coordinated by the His122-His123-Asp191 (HHD) motif. The binding mode of modeled 3IPM and SAM (*grey* carbon atoms) was predicted with Autodock Vina based on a fixed geometry of protein residues and SAM (32, 33). The C1-carboxy group interacts with Arg22 and calcium. *Dots* represent interactions between 3IPM and the metal ion, cosubstrate, and protein residues. The *double-arrow* shows the methyl transfer trajectory.

To elucidate the molecular mechanism of O-methyltransferases in β-lactone biosynthesis, we solved high-resolution structures of BelI and CysG. Both proteins were heterologously expressed in *Escherichia coli* and purified *via* NiNTA-affinity and size exclusion chromatography. Full-length BelI crystallized in space group F222 with defined electron density observed for residues 17 to 228 (PDB ID: 9FCE). In contrast, N-terminal truncation of CysG (CysG^ΔN16^) was essential to achieve diffracting crystals in space group P42_1_2, with electron density mapped for residues 17 to 229 (PDB ID: 9FCD). As expected, BelI as well as CysG^ΔN16^ adopt the class I methyltransferase Rossmann fold, characterized by a core domain of a seven-stranded β-sheet flanked by α-helices (Fig. 2, *A* and *B*) (28, 29). Both transferases share a remarkable structural similarity as shown in Figure 2*C* (backbone root mean square deviation (rmsd) = 0.6 Å, 96% C^α^-atoms, 66% sequence identity). Therefore, we introduced a uniform numbering based on their sequence alignment (Fig. S1). A structure homology search using the DALI server (30) confirmed significant similarities with other SAM-dependent methyltransferases, with the putative methyltransferase-MM_2633 from *Methanosarcina mazei* (PDB ID: 3DTN) emerging as the closest match in the RCSB database (Z-score = 32.0; sequence identity 40%). However, the unique N-termini of BelI and CysG^ΔN16^ are indicative of their specialized roles in substrate recognition (see below).

The crystal structures of CysG^ΔN16^ in complex with *S*-adenosylhomocysteine (SAH) and BelI bound to SAM were solved at 1.5 Å and 1.95 Å, respectively. The 2F_o_-F_c_ electron density maps depict the cofactors at atomic resolution and illustrate similar interactions with protein residues of either BelI or CysG. Specifically, the terminal carboxyl and amino groups of SAH/SAM are forming hydrogen bonds with Tyr33, Gly54, and residue 118. The ribose unit is coordinated *via* Asp77, while the adenine portion is stabilized by Asp102, residue 103, and cation-π interactions with Arg78 (Fig. S2, *A* and *B*).

Intriguingly, the crystal structures of CysG^ΔN16^ and BelI revealed a His122-His123-Asp191 (HHD) motif with additional strong positive electron density, indicative of a divalent metal ion (Fig. 2*D*). The absence of anomalous signals in X-ray fluorescence experiments conducted with the crystals suggests calcium or magnesium as likely candidates. Notably, the complex depicts octahedral metal coordination with bond lengths between 2.0 and 2.4 Å and bond angles, which are consistent with the binding profile of calcium (Ca^2+^) (Fig. S2*C*) (31).

Despite extensive crystallization efforts, defined electron density for the substrates 3-sec-butylmalate (3SBM) and three-isopropylmalate (3IPM) were not detectable in the active sites of BelI and CysG^ΔN16^. Molecular analysis indicates that a flexible N-terminus in both enzymes likely plays a key role in substrate binding. Furthermore, phenylalanine in BelI and tyrosine in CysG, at position 18, act as gatekeepers (see below) and can either adopt an open or closed conformation important for catalysis. These findings are consistent with previous reports on other OMTases (29). We thus conclude that the rigidity of the gatekeeper in its closed conformation, potentially due to crystal packing, may hinder ligand binding even at high concentrations up to 10 mM. Yet, our atomic-resolution structures combined with the structural data of the active site composition and identification of the methylation products provide valuable parameters for computational modeling. Using the AutoDock Vina tool (32, 33), we analyzed substrate coordination within the active sites of BelI and CysG^ΔN16^. Indeed, ligand docking calculations predicted identical binding modes for 3IPM and 3SBM in both enzymes and suggested an S_N_2-like reaction mechanism (34). Specifically, the C1-carboxyl group of each substrate interacts with Arg22 and the divalent metal ion, while the C4-carboxyl group forms hydrogen bonds with His167 and Arg187. In addition, the aliphatic side chains of 3IPM and 3SBM are anchored within an apolar specificity pocket formed by Ile26, Leu119, and Phe220 (Figs. 2*D* and S3).

**Figure 3:**
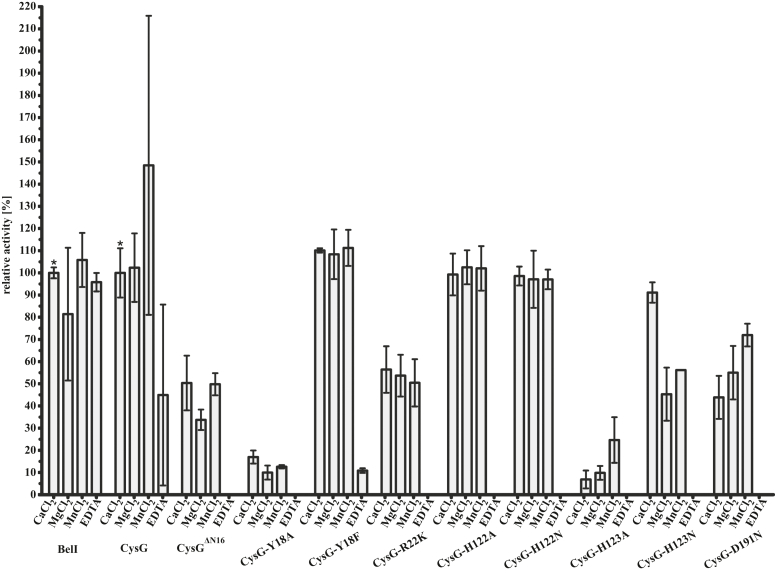
**HPLC-MS Activity Assays of Enzymatic Reactions with Divalent Metal Ions.** Activity assays for BelI, CysG^fl^, CysG^ΔN16^, and CysG variants, either showing conversion of 3IPM to 1-methyl-3IPM or 3SBM to 1-methyl-3-SBM. The enzymatic activity was calculated semi-quantitatively by comparing substrate and product intensities within the same measurement (Fig. S7). Percentage activities were referenced to the mean of the wild-type enzymes in the presence of calcium (∗). Data represent mean ± standard deviation from assays performed in triplicate.

Next, we aimed to elucidate molecular insights into the reaction process in BelI and CysG, focusing on proximity-driven, acid-base, and metal-dependent catalytic principles (5, 6). Therefore, we conducted high-performance liquid chromatography coupled with mass spectrometry (HPLC-MS) for activity assays. While 3IPM, the substrate for CysG, is commercially available, we synthesized 3SBM to study BelI activity (27). Semiquantitative monitoring of reaction products in the presence of Ca^2+^ of BelI (Fig. S4*A*) and full-length CysG (CysG^fl^) (Fig. S5*A*), combined with structural data, revealed striking similarities in function and catalysis between both transferases. Due to the high crystallization tendency of CysG^ΔN16^, subsequent mutagenesis experiments were focused on this variant. This approach enabled the production of both full-length and N-terminal truncated mutants in high purity (Fig. S6), which were characterized in activity assays (Figs. 3 and S7) and used for structural studies (Fig. S8). Structure-activity relationship analysis in the presence of Ca^2+^ identified residue 18 as a gatekeeper: the CysG^fl^-Y18F variant is fully active (Fig. S9*A*), whereas the CysG^fl^-Y18A mutant retains only 17% of the wild-type (wt) activity (Fig. S10*A*). Moreover, Arg22 was found to be important for cosubstrate binding, with the CysG^fl^-R22K mutant displaying a 44% reduction in activity compared to wt (Fig. S11*A*). Interestingly, the crystal structure of the corresponding CysG^ΔN16^-R22K variant (PDB ID: 9FCY) maintains SAM coordination, though with diffuse electron density (Fig. S8*C*). These results indicate that effective catalysis in BelI and CysG depends on specific substrate-protein interactions within the active site, challenging the notion of pure proximity- or acid-base-driven catalysis.

To investigate the metal dependency of both enzymes, we performed HPLC-MS-based activity assays with various divalent cations (Figs. 3, S4, S5, S7, S9–S18). We selected calcium, magnesium, and manganese due to their similar but distinct properties regarding ion radii, complex stability, and increasing electronegativity as well as Lewis acidity from Ca^2+^ to Mn^2+^. Activities were referenced to wt enzymes in the presence of Ca^2+^, respectively (Figs. S4*A* and S5*A*). Surprisingly, wild-type BelI and CysG^fl^ retained activity when incubated with EDTA during the reaction (Figs. S4*D* and S5*D*). Therefore, we conducted structure-based mutagenesis by introducing non-conservative alanine residues in the HHD motif to further explore the metal-binding site. As expected, CysG^fl^-H123A was much less active (Fig. S12), with significant rearrangements at the metal-binding site observed in the crystal structure of the corresponding CysG^ΔN16^-H123A variant (PDB ID: 9FCQ) (Fig. S8*F*). In contrast, the CysG^fl^-H122A variant remained active across diverse metal ions but was inactivated by EDTA (Fig. S13). Therefore, His122 is involved in metal stabilization without affecting the interaction between protein and substrate. Conservative asparagine replacements in the HHD motif resulted in CysG variants that remained active. CysG^fl^-D191N showed a 56% decrease in substrate turnover (Fig. S14), while CysG^fl^-H122N (Fig. S15) and CysG^fl^-H123N (Fig. S16) were fully active compared to wt but were inactivated with EDTA. Strikingly, the CysG^fl^-H123N variant showed 91% of the original catalytic activity with Ca^2+^ (Fig. S16*A*), whereas Mg^2+^ (Fig. S16*B*) and Mn^2+^ (Fig. S16*C*) resulted in only 50% residual activity. Notably, the N-terminally truncated CysG^ΔN16^ variant was active with metal ions available but inactive in the presence of EDTA (Fig. S17). This prompted us to solve the metal-free structure for atomic insights (PDB ID: 9G0K). Indeed, the 2F_o_-F_c_ electron density map displayed the structural integrity of the enzyme in a metal-free state (Fig. S8*I*). Thus, we conclude that the divalent metal ion is crucial for the catalytic function of CysG rather than for structural stability. Taken together, we have elucidated a previously unknown metal-dependent mechanism of OMTases, highlighting the intricate interplay between divalent metal ions and enzyme activity that is fundamental to SAM-mediated methyl transfer in these catalysts.

## Discussion

Our structure-activity relationship study reveals molecular insights into the catalysis of the O-methyltransferases CysG and BelI from the cystargolide and belactosin biosynthetic pathways. Notably, metal-dependent mechanisms are most prominent in phenolic OMTases, in which the metal is commonly coordinated by hydroxyl and carboxyl ligands (35, 36). For example, caffeoyl coenzyme A 3-OMTase incorporates Ca^2+^ in the active site, forming a reactive oxyanion intermediate (37), whereas the methylation of antibiotic mycinamicins shows a mixed base-catalyzed and Mg^2+^-dependent mechanism (38). Here, we demonstrated that CysG and BelI operate *via* a divalent metal-dependent mechanism utilizing a His-His-Asp (HHD) metal-binding motif, a unique feature so far not described in other OMTases (35, 36, 37, 38). The HHD-motif enables regiospecific C1-carboxy group methylation of their respective malic substrates. Analysis of the complex geometry in high-resolution structures, X-ray fluorescence measurements, as well as metal-dependent activity assays with the CysG^fl^-H123N variant indicate Ca^2+^ as the intrinsically bound metal ion. The activity assays revealed that the calcium is tightly bound in the full-length wild-type enzymes, as it can only be removed in the presence of EDTA or replaced by other metal ions if either the N-terminus is deleted, the gating residue is mutated, or the metal binding motif is disrupted.

In conclusion, CysG and BelI are methyltransferases whose metal dependency is integral to their function. These transferases might represent an evolutionary adaptation tailored to their unique biochemical roles and differ from previously characterized OMTases. The methylation, although absent in the final natural products due to hydrolysis by a specific enzyme, likely serves as a protection group strategy that is essential for the initial β-lactone warhead formation in cystargolides and belactosins. Similar to organic synthesis routes, the methylation of the C1-carboxy group might prepare for the activation of the C4-carboxy group by reducing electrostatic repulsion in the sterically demanding lactonization reaction (26). Furthermore, in cystargolide biosynthesis, the formation of the methyl ester by CysG could prevent decarboxylation and the entry of 3IPM into the primary leucine anabolic pathway.

## Experimental procedures

### General experiments

Chemicals, micro-, and molecular biological agents were acquired from standard commercial sources. *E. coli* strains were grown in a lysogeny broth (LB) medium supplemented with appropriate antibiotics. DNA isolation and manipulations were carried out according to standard methods for *E. coli*.

### CysG and BelI enzyme assay conditions

A standard enzyme assay contained 1 mM substrate, 1 mM SAM, and 5 μM BelI, CysG, or mutant constructs in a total of 50 μl reaction volume. The enzyme assay buffer (50 mM Tris-HCl, 20 mM KCl, 10% (v/v) glycerol) was completed by the addition of different divalent metal ions or EDTA to a final concentration of 20 mM, respectively. The assay was started by the addition of 3IPM for CysG and 3SBM for BelI, and the reaction was run for 3 h at 30 °C. The reaction was stored at −20 °C until subjected to LC-MS analysis or purification. Negative controls did not contain either substrate, SAM, or enzyme. Enzyme assays were analyzed by LC-MS, performed on an Agilent Infinity 1260 II System. Samples (10 μl) were injected onto a reverse phase column (ReproSil 100 C18, particle size 3 μm, pore size 100 Å, 100 × 3 mm) at a flow rate of 0.3 ml/min and a linear gradient of solvent B from t_0_ = 10% to t_20_ = t_30_ = 100% and t_31_ = t_40_ = 20% (solvent A: water with 0.1% formic acid, solvent B: MeCN with 0.1% formic acid). The column was heated to 30 °C. Ionization and mass analysis were performed on a Bruker AmaZon SL system by ESI (negative ionization) with a capillary voltage of 4.5 kV and a drying gas temperature of 125 °C. Enzymatic activity was semi-quantitatively calculated by comparing the intensities of the substrate and product within each measurement. A comparison of assays performed without enzymes present in the reaction mix showed only little difference between reactions performed with different metal ion-containing buffers (Fig. S18). This applies to both 3IPM and 3SBM and supports the conclusion that variation in extraction efficiencies is minor.

### Cloning and protein expression

The synthetic gene fragments optimized for *E. coli* codon usage of BelI (GenBank ARO49577.1) and CysG (GenBank ARO49565.1) (Table S1) were cloned into a pETDuet expression vector modified to encode an N-terminal His_6_-SUMO tag using NEBuilder HiFi DNA Assembly following the manufacturer’s instructions. Mutagenesis was conducted following the instructions of the Q5 Site-Directed Mutagenesis Kit (NEB) with primers according to Table S2. Correct insertion was verified by Sanger sequencing (Eurofins genomics). *E. coli* BL21(DE3) cells were transformed by electroporation and grown in glass shake flasks containing 2 L lysogenic broth with 100 mg/L ampicillin at 37 °C. After reaching an optical density measured at a wavelength of 600 nm (OD_600_) of 0.6 to 0.8, flasks were stored at 4 °C for 30 minutes before adding isopropyl-β-*D*-1-thiogalactopyranoside to a final concentration of 0.5 mM to induce protein expression. After incubation with shaking overnight at 20 °C, cells were harvested by centrifugation, washed with 0.9% (w/v) sodium chloride, and cell pellets were stored at −20 °C until further use.

### Protein purification protocol

*E. coli* pellets were dissolved in buffer A (100 mM Tris/HCl pH 7.5, 200 mM NaCl, 20 mM imidazole, 2 mM 2-mercaptoethanol) and lysed by sonication (Branson Digital Sonifier 250). After centrifugation (40,000*g*, 4 °C, 30 min), the supernatant was applied onto a 5 ml HisTrap HP column at a flow rate of 5 ml/min with an ÄKTA Pure system (Cytiva), previously equilibrated with buffer A. After washing with buffer A, the protein of interest was eluted by applying a linear gradient from 0 to 100% buffer B (100 mM Tris/HCl pH 7.5, 200 mM NaCl, 500 mM imidazole, 2 mM 2-mercaptoethanol) in 10 column volumes. Fractions containing protein were pooled and supplemented with SUMO-protease to remove the His_6_-SUMO-tag and dialyzed overnight at 4 °C against buffer C (20 mM Tris/HCl pH 7.5, 200 mM NaCl, 2 mM 2-mercaptoethanol). HisTrap affinity chromatography was repeated and the flow-through was concentrated to 4 ml using Amicon Ultra-15 centrifugal filters (30,000 MWCO). Centrifugation (16,000*g*, 4 °C, 15 min) removed residual protein aggregates, and the supernatant was used for size exclusion chromatography with a HiLoad Superose 6 pg 16/600 column in buffer D (20 mM Tris/HCl pH 7.5, 200 mM NaCl, 2 mM dithiothreitol) at 1.0 ml/min. Purity was assigned by Coomassie-stained SDS-PAGE analysis, pure fractions were pooled and concentrated to 10 to 15 mg/ml.

### Crystallization and structure determination of CysG^ΔN16^ and BelI

Either 10 mg/ml of (mutant) CysG^ΔN16^ or 15 mg/ml of BelI were mixed with 2 mM of SAH or SAM, and optionally 2 to 10 mM of 3IPM or 3SBM, respectively, were added. After incubation at 4 °C for 1 h, the precipitant was removed by centrifugation (16,000*g*, 4 °C, 15 min) and the solution was subsequently used for crystallization trials. Metal-free CysG^ΔN16^ was prepared for crystallization trials by incubation with 5 mM EDTA for 2 h at 4 °C. Stepwise dialysis against EDTA-free buffer resulted in the metal-free enzyme used for crystallization trials in the presence of 2 mM EDTA. Sparse-matrix screens were set up with a drop ratio of 0.2 μl + 0.2 μl, 0.2 μl + 0.1 μl, or 0.3 μl + 0.1 μl at 20 °C. The reservoir conditions for the best diffracting crystals are listed in Table S3. Crystals grew within 1 week and were cryo-protected with 20 to 30% ethylene glycol before vitrification in liquid nitrogen. Data sets were recorded at the beamline X06SA at the Swiss Light Source in Villingen, Switzerland, and beamline P13 (PETRA III) at the DESY (Hamburg, Germany).

The XDS software package was used for initial data processing and scaling (39). Details of data collection and analysis are listed in Tables S4–S7. All further steps for structure solution were performed using programs of the CCP4 software package (40). Conventional crystallographic rigid body, positional, and temperature factor refinements were carried out with REFMAC5 (41) using coordinates of 3DTN (https://www.rcsb.org/) as starting models. For model building, the programs SYBYL and COOT (42) were used. The final coordinates yielded excellent R factors, as well as geometric bond and angle values. Coordinates were confirmed to fulfill the Ramachandran plot and have been deposited in the RCSB.

### Computation of docking structures

Structural models of proteins (BelI, CysG^ΔN16^) and ligands (3SBM, 3IPM) were prepared for docking calculations using AutoDockTools 1.5.7 (ADT). Grid boxes for the docking simulations were defined using ADT. Charges to metal ions were assigned to +2. Parameters were maintained at the default configuration. Subsequently, dockings were performed with the AutoDock Vina tool based on a fixed geometry of the protein and SAM. PyMol was used to analyze the binding modes of 3SBM and 3IPM to BelI and CysG^ΔN16^, respectively (32, 33).

### Chemical synthesis

Standard laboratory glassware and equipment were used to carry out all experiments. All chemicals and solvents were purchased from Sigma Aldrich, Carl Roth, or Acros and used without further purification. For the removal of volatiles, a BUCHI Rotavapor R-215 with a Thermo Haake K10/DC10 cooling system was used. Purifications of chemical products were performed on a BUCHI Pure C-815 Flash Chromatography System using appropriate FlashPure EcoFlex Silica cartridges. Analytical thin-layer chromatography (TLC) was performed on aluminium-coated TLC silica gel plates (silica gel 60, F254, Merck). For visualization, ultra-violet irradiation (λ = 254 nm) and/or staining using potassium permanganate solution (3.0 g KMnO_4_, 20.0 g K_2_CO_3,_ and 5 ml 5% NaOH in 300 ml H_2_O) with subsequent heat treatment were used.

Mass spectrometry measurements were recorded on an Agilent Infinity II 1290 LCMS system equipped with a Single Quadrupol MS (6100 series, AJS ion source) and a C18-column (Poroshell 120 EC-C18, 1.9 μm, 2.1 × 100 mm) using a 5 to 95% H_2_O-acetonitrile (ACN) gradient over 10 min with a flow rate of 0.4 ml/min. Solvents were supplemented with 0.1% formic acid. The applied scan range was m/z 100 to 2000 in positive and negative mode.

Nuclear magnetic resonance (NMR) spectroscopy experiments were conducted using a Bruker AVHD-400 instrument at 303 K operating at 400 MHz (^1^H) and 101 MHz (^13^C), respectively. Chemical shifts are reported in part per million (ppm) and referred to the solvent signal. The coupling constants are given in Hz and the multiplicities of the ^1^H NMR are defined as s-singlet, d-doublet, dd-doublet of doublets, t-triplet, m-multiplet.

## 3-sec-butylmalate

Chemical synthesis of 3-sec-butylmalate was carried out according to our previous protocol (27). In brief summary, lithium bis(trimethylsilyl)amide solution (1 M in THF, 23.13 ml, 23.1 mmol, 2.2 eq.) was cooled to −78 °C. Diethyl malate (2.0 g, 10.5 mmol, 1.0 eq.), dissolved in 10 ml THF, was added dropwise over 30 min and the reaction mixture was stirred at −78 °C for 1 h under an atmosphere of nitrogen and at 20 °C for an additional hour. Afterward, the reaction mixture was cooled to −78 °C, and two-iodobutane (3.27 ml, 28.4 mmol, 2.7 eq.) was added over 10 min and stirred for 1 h at −78°C. The reaction mixture was stirred overnight while allowing to reach 20 °C. The reaction was quenched with 200 ml saturated ammonium chloride solution and extracted with ethyl acetate (3 × 100 ml). The combined organic phases were washed with brine (2 × 30 ml), dried over Na_2_SO_4_, and concentrated under reduced pressure. The crude product was purified with a BUCHI Pure C-815 Flash Chromatography System using a FlashPure EcoFlex Silica 12g cartridge using ethyl acetate and hexane (1:2) as solvents. 3-sec-butylmalate diethylester (693 mg, 2.8 mmol, 27%) was obtained as a pale-yellow oil. In a subsequent step, diethyl 3-sec-butylmalate diethylester (200 mg, 0.8 mmol, 1.0 eq.) was dissolved in 2.7 ml methanol at 0 °C under an atmosphere of nitrogen. Potassium hydroxide (200 mg, 3.6 mmol, 4.4 eq.) was added in one portion and stirred at 0 °C for 2 h. Volatiles were removed under reduced pressure and 3-sec-butylmalate (134 mg, 0.7 mmol, 87%) was obtained as a pale-yellow solid.

^1^H NMR (400 MHz, MeOD) *δ* [ppm] = 4.07 (d, J = 2.8 Hz, 1H), 2.69–2.54 (m, 1H), 2.43–2.38 (m, 1H), 1.29–1.16 (m, 2H), 1.00 (d, J = 6.8 Hz, 3H), 0.93 (t, J = 7.5 Hz, 3H).

^13^C NMR (101 MHz, MeOD) *δ* [ppm] = 181.6, 180.4, 73.2, 58.1, 35.0, 27.4, 18.0, 11.4.

MS (-ESI): calc. for C_8_H_13_O_5_ [M-H]^−^: 189.08; found 189.1.

## Data availability

All data presented are included within the article.

## Supporting information

This article contains supporting information (31, 32, 33, 43).

## Conflict of interest

The authors declare no conflicts of interest with the contents of the article.
