# Supplementary material for: Deciphering the SAM- and metal-dependent mechanism of O-methyltransferases in cystargolide and belactosin biosynthesis: A structure–activity relationship study
Source: J Biol Chem. 2024 Aug 8;300(9):107646. doi: 10.1016/j.jbc.2024.107646 (PMC11408123; doi:10.1016/j.jbc.2024.107646)
Supplement: Supplemental Tables S1–S7 and Figure S1–S18 [file mmc1.docx]

**Supporting Information**

**Title**

Deciphering the SAM- and Metal-Dependent Mechanism of O-Methyltransferases in Cystargolide and Belactosin Biosynthesis: A Structure-Activity Relationship Study

**Authors**

Wolfgang Kuttenlochner^1*^, Patrick Beller^2^, Leonard Kaysser^3^, Michael Groll^1*^

^1^ Department of Bioscience, Center for Protein Assemblies (CPA), TUM School of Natural Sciences, Technical University of Munich, Garching, Germany

^2^ Department of Pharmaceutical Biology, Pharmaceutical Institute, University of Tübingen, Tübingen, Germany

^3^ Department of Pharmaceutical Biology, Institute for Drug Discovery, University of Leipzig, Leipzig, Germany

*For correspondence: Wolfgang Kuttenlochner [wolfgang.kuttenlochner@tum.de](mailto:wolfgang.kuttenlochner@tum.de), Michael Groll [michael.groll@tum.de](mailto:michael.groll@tum.de)

**Table S1|** Codon-optimized gene sequences used in this work.

| Gene | Sequence 5’ 🡪 3’ |
| --- | --- |
| *belI* | GCTCAGACCTTCGAAATCAAAGGTAACGACCTGTGGGACCCGACCACCTTCGACGCTCTGCGTCGTCAGCTGATCCCGTCTTTCGACCTGATCTACGAAGCTGCTGTTCGTACCGTTGCTGCTACCGTTCCGACCGCTCCGCGTGTTCTGGACCTGGGTGCTGGTACTGGTCTGCTGTCTGCTGCTATCCTGCGTGAACTGCCGGACTCTGAAGTTGTTCTGGTTGACCGTTCTGAACTGATGCTGACCCAGGCTCGTGGTCGTTTCGCTTCTCAGGACGGTGTTACCGTTCAGACCGGTGACCTGACCGACCCGCTGCCGGAAGGTGGTTTCGACGCTGTTGTTTCTGGTCTGGCTATCCACCACCTGTCTCACACCGGTAAACGTGACCTGTTCCGTCGTATCCGTGAAGCTCTGCGTCCGGGTGGTGTTTTCGTTAACGTTGAACAGGTTCAGGGTCCGCTGCCGCACCTGGAATCTCTGTACGACTCTCAGCACGAACTGCACGTTATCCGTGAACAGGCTCCGGCTCACGAATGGGCTGCTGGTCGTGAACGTATGAAATTCGACGTTTGCATCGACCTGGAAACCCAGCTGCAATGGCTGCGTGACGCTGGTTTCCGTTCTGTTGACTGCCTGGCTAAAGACTTCCGTTTCGCTACCTACGCTGGTTGGGTTTCT |
| *cysG* | TCTCAGACCGCTTCTCCGCGTATCGAACTGTGGAACCCGGAAACCTACGACGCTCTGCGTCGTCAGCTGATCCCGTCTTTCGACCTGCTGTACGGTTCTGCTGTTTCTGTTGTTGCTATGTCTGTTCCGGCTACCGCTCGTATCCTGGACCTGGGTGCTGGTACTGGTCTGCTGGGTGCTGCTCTGCGTGAACGTCTGCCGGACGCTGAACTGCTGCTGCAAGACCGTTCTCAGGCTATGCTGGAACAGGCTCGTCAGCGTTTCGCTGACGACGACCAGGTTGCTATCCGTGTTGCTGACCACCTGGACGAACTGCCGGCTGGTCCGTTCGACGCTGTTGTTTCTGCTCTGTCTATCCACCACCTGGAACACCAGGACAAACAGGACCTGTTCACCCGTATCCGTAAAATCCTGCGTCCGGGTGGTATCTTCGTTAACGTTGAACAGGTTCTGGCTCCGACCTCTGAACTGGAAAAAATGTACGACCGTCAGCACGAAGCTCACGTTCTGGCTTCTGACACCCCGGCTGAAGAATGGGCTGCTGGTCGTGAACGTATGAAACACGACATCCCGATCGACGTTGAAACCCAGATCCAGTGGCTGCGTGACGCTGGTTTCACCACCGCTGACTGCCTGGCTAAAGACTGGCGTTTCGCTACCTACGCTGGTTGGAACGGTTCT |

**Table S2|** Oligonucleotides used in this study.

| Oligonucleotide | Sequence 5’ 🡪 3’ |
| --- | --- |
| BelI_pETDuet_SUMO.FOR | ACAGATCGGTGGATCCGCTCAGACCTTC |
| BelI_pETDuet_SUMO.REV | GCCGTGTACAATACGATTAAGAAACCCAACCAGCGTAGG |
| CysG_pETDuet_Sumo.FOR | ACAGATCGGTGGATCCTCTCAGACCGCT |
| CysG_pETDuet_Sumo.REV | GCCGTGTACAATACGATTAAGAACCGTTCCAACCAGCG |
| CysG_dN16_pETDuet_Sumo.FOR | ACAGATCGGTGGATCCACCTACGACGCTCTGCGTCG |
| Q5_CysG_Y18A.FOR | GGAACCCGGAAACCGCAGACGCTCTGCGTCGT |
| Q5_CysG_Y18F.FOR | GGAACCCGGAAACCTTCGACGCTCTGCGTCGT |
| Q5_CysG_Y18.REV | ACAGTTCGATACGCGGAGAAGC |
| Q5_mut_CysG_R22K.FOR | CGACGCTCTGAAACGTCAGCTGATC |
| Q5_mut_CysGfl_R22.REV | TAGGTTTCCGGGTTCCACAG |
| Q5_mut_CysGdN16_R22.REV | TAGGTGGATCCACCGATCTGTTC |
| Q5_CysG_H122A.FOR | TCTGTCTATCGCCCACCTGGAACACC |
| Q5_CysG_H122N.FOR | TCTGTCTATCAACCACCTGGAACACC |
| Q5_CysG_H122.REV | GCAGAAACAACAGCGTCG |
| Q5_CysG_H123A.FOR | GTCTATCCACGCGCTGGAACACCAGGAC |
| Q5_CysG_H123N.FOR | GTCTATCCACAACCTGGAACACCAGGAC |
| Q5_CysG_H123.REV | AGAGCAGAAACAACAGCG |
| Q5_CysG_D191N.FOR | TATGAAACACAACATCCCGATCGACG |
| Q5_CysG_D191N.REV | CGTTCACGACCAGCAGCC |
| pETDuet_SUMO_Vector.FOR | TCGTATTGTACACGGCCGC |
| pETDuet_SUMO_Vector.REV | GGATCCACCGATCTGTTCACG |

**Table S3|** Crystallization conditions for BelI, CysG^ΔN16^, and its mutants. *constructs names are given according to the used enumeration throughout this manuscript and refer to table S1.

| Construct* | Additives | Crystallization condition | Droplet volume (protein/screen) | PDB ID |
| --- | --- | --- | --- | --- |
| BelI | 2 mM SAM | 0.2 M Lithium chloride 0.1 M TRIS pH 8, 20%(w/v) PEG 8000 | 0.2 μL/0.1 μL | 9FCE |
| CysG^ΔN16^ | 5 mM 3IPM, 5 mM SAH | 2.0 M Ammonium sulfate, 0.1 M HEPES sodium salt pH 7.5, 2 %(v/v) PEG 400 | 0.2 μL/0.2 μL | 9FCD |
| CysG^ΔN16^ | 5 mM SAM | 0,1M HEPES pH 7.1, 0.2 M sodium chloride, 26 % PEG4000 | 0.2 μL/0.2 μL | 9FCL |
| CysG ^ΔN16^-R22K | 5mM IPM, 2mM SAH | 0,1M HEPES pH 7.1, 0.2 M sodium chloride, 20 % PEG4000 | 0.2 μL/0.2 μL | 9FCY |
| CysG^ΔN16^-H122A | 5 mM 3IPM, 5 mM SAH | 0.2 M Sodium chloride 0.1 M HEPES pH 7.5, 25 %(w/v) PEG 3350 | 0.2 μL/0.1 μL | 9FCU |
| CysG^ΔN16^-H122N | 5 mM 3IPM, 5 mM SAH | 0.2 M Sodium chloride, 0.1 M HEPES pH 7.5, 25%(w/v) PEG 4000 | 0.2 μL/0.1 μL | 9FCX |
| CysG^ΔN16^-H123A | 5 mM 3IPM, 5 mM SAH | 0.02 M Magnesium chloride, 0.1 M HEPES pH 7.5, 22 %(w/v) Sodium Polyacrylate 5100 | 0.2 μL/0.1 μL | 9FCQ |
| CysG^ΔN16^-H123N | 5 mM 3IPM, 5 mM SAH | 0.02 M Magnesium chloride, 0.1 M HEPES pH 7.5, 22 %(w/v) Sodium Polyacrylate 5100 | 0.3 μL/0.1 μL | 9FCS |
| CysG^ΔN16^-D191N | 5 mM 3IPM, 5 mM SAH | 0.24 M Sodium malonate pH 7.0, 20 %(w/v) PEG 3350 | 0.2 μL/0.2 μL | 9FD3 |
| CysG^ΔN16^  (metal-free) | 5 mM 3IPM, 5 mM SAH,  2mM EDTA | 0.2 M Lithium chloride 0.1 M TRIS pH 8, 20%(w/v) PEG 8000 | 0.2 μL/0.2 μL | 9G0K |

**Table S4|** Crystallographic data collection and refinement statistics.

|  | **CysG^ΔN16^:SAH** | **CysG^ΔN16^:SAM** | **BelI:SAM** |
| --- | --- | --- | --- |
|  |  |  |  |
| **Crystal parameters** |  |  |  |
| Space group | P42_1_2 | P42_1_2 | F222 |
| Cell constants | a = 101.2 Å  b = 101.2 Å  c = 49.8 Å | a = 101.5 Å  b = 101.5 Å  c = 49.9 Å | a = 81.4 Å  b = 165.0 Å  c = 165.7 Å |
| molecules / AU^a^ | 1 | 1 | 2 |
|  |  |  |  |
| **Data collection** |  |  |  |
| Beam line | X06SA, SLS | X06SA, SLS | X06SA, SLS |
| Wavelength (Å) | 1.0 | 1.0 | 1.0 |
| Resolution range (Å)^b^ | 30-1.5 (1.6-1.5) | 30-1.75 (1.85-1.75) | 30-1.95 (2.05-1.95) |
| No. observations | 181865 | 130001 | 151452 |
| No. unique reflections^c^ | 40256 | 26735 | 28727 |
| Completeness (%)^b^ | 96.0 (96.4) | 99.4 (99.8) | 95.1 (97.4) |
| R_merge_ (%)^b, d^ | 5.7 (60.5) | 6.3 (65.5) | 4.2 (57.3) |
| I/σ (I)^b^ | 15.3 (3.5) | 13.1 (2.3) | 15.9 (2.2) |
|  |  |  |  |
| **Refinement (REFMAC5)** |  |  |  |
| Resolution range (Å) | 30-1.5 | 30-1.75 | 30-1.95 |
| No. refl. working set | 38239 | 25391 | 36781 |
| No. refl. test set | 2012 | 1336 | 1936 |
| No. non hydrogen | 1875 | 1834 | 3486 |
| No. of ligand atoms | 26 | 27 | 54 |
| No. of calcium ions | 1 | 1 | 2 |
| Solvent (H_2_O, ions, buffer) | 173 | 138 | 162 |
| R_work_/R_free_ (%)^e^ | 14.6/17.1 | 15.7/19.9 | 17.0/20.7 |
| r.m.s.d. bond (Å) / angle (°)^f^ | 0.005/1.2 | 0.004/1.2 | 0.004/1.1 |
| Average B-factor (Å^2^) | 20.0 | 32.0 | 50.3 |
| Ramachandran Plot (%)^g^ | 98.6/1.4/0 | 98.6/1.4/0 | 98.5/1.5/0 |
|  |  |  |  |
| PDB accession code | 9FCD | 9FCL | 9FCE |

^[a]^ Asymmetric unit

^[b]^ The values in parentheses for resolution range, completeness, R_merge_ and I/σ (I) correspond to the highest resolution shell

^[c]^ Data reduction was carried out from a single crystal. Friedel pairs were treated as identical reflections

^[d]^ R_merge_(I) = Σ_hkl_Σ_j_ | I(hkl)_j_ - <I(hkl)> | / Σ_hkl_ Σ_j_ I(hkl)_j_, where I(hkl)_j_ is the j^th^ measurement of the intensity of reflection hkl and <I(hkl)> is the average intensity

^[e]^ R = Σ_hkl_ | |F_obs_| - |F_calc_| |/Σ_hkl_ |F_obs_|, where R_free_ is calculated without a sigma cut off for a randomly chosen 5% of reflections, which were not used for structure refinement, and R_work_ is calculated for the remaining reflections

^[f]^ Deviations from ideal bond lengths/angles

^[g]^ Percentage of residues in favored / allowed / outlier region

**Table S5|** Crystallographic data collection and refinement statistics.

|  | **CysG^ΔN16^-R21K:SAH** | **CysG^ΔN16^-H121A:SAH** | **CysG^ΔN16^-H121N:SAH** |
| --- | --- | --- | --- |
|  |  |  |  |
| **Crystal parameters** |  |  |  |
| Space group | P2_1_ | P2 | P4_2_2_1_2 |
| Cell constants | a = 50.0 Å  b = 106.2 Å  c = 99.0 Å  β = 90.25 ° | a = 100.8 Å  b = 49.7 Å  c = 102.5 Å  β = 90.1 ° | a = 99.9 Å  b = 99.9 Å  c = 98.3 Å |
| molecules / AU^a^ | 4 | 4 | 2 |
|  |  |  |  |
| **Data collection** |  |  |  |
| Beam line | X06SA, SLS | X06SA, SLS | X06SA, SLS |
| Wavelength (Å) | 1.0 | 1.0 | 1.0 |
| Resolution range (Å)^b^ | 30-1.85 (1.95-1.85) | 30-1.85 (1.95-1.85) | 30-1-8 (1.9-1.8) |
| No. observations | 266517 | 244434 | 212195 |
| No. unique reflections^c^ | 87037 | 85811 | 45416 |
| Completeness (%)^b^ | 98.8 (99.5) | 98.3 (98.8) | 97.3 (99.8) |
| R_merge_ (%)^b, d^ | 5.4 (57.5) | 9.4 (68.3) | 7.5 (62.4) |
| I/σ (I)^b^ | 12.3 (2.4) | 9.0 (2.6) | 11.7 (2.4) |
|  |  |  |  |
| **Refinement (REFMAC5)** |  |  |  |
| Resolution range (Å) | 30-1.85 | 30-1.85 | 30-1.8 |
| No. refl. working set | 82670 | 81503 | 43132 |
| No. refl. test set | 4351 | 4290 | 2270 |
| No. non hydrogen | 7035 | 7175 | 3674 |
| No. of ligand atoms | 40 | 104 | 52 |
| No. of calcium ions | 4 | 0 | 0 |
| Solvent (H_2_O, ions, buffer) | 353 | 391 | 264 |
| R_work_/R_free_ (%)^e^ | 14.9/19.6 | 19.2/23.1 | 15.9/20.4 |
| r.m.s.d. bond (Å) / angle (°)^f^ | 0.004/1.2 | 0.003/1.2 | 0.003/1.1 |
| Average B-factor (Å^2^) | 34.5 | 22.3 | 27.5 |
| Ramachandran Plot (%)^g^ | 98.3/1.7/0 | 98.9/1.1/0 | 98.8/1.2/0 |
|  |  |  |  |
| PDB accession code | 9FCY | 9FCU | 9FCX |

^a]^ Asymmetric unit

^[b]^ The values in parentheses for resolution range, completeness, R_merge_ and I/σ (I) correspond to the highest resolution shell

^[c]^ Data reduction was carried out from a single crystal. Friedel pairs were treated as identical reflections

^[d]^ R_merge_(I) = Σ_hkl_Σ_j_ | I(hkl)_j_ - <I(hkl)> | / Σ_hkl_ Σ_j_ I(hkl)_j_, where I(hkl)_j_ is the j^th^ measurement of the intensity of reflection hkl and <I(hkl)> is the average intensity

^[e]^ R = Σ_hkl_ | |F_obs_| - |F_calc_| |/Σ_hkl_ |F_obs_|, where R_free_ is calculated without a sigma cut off for a randomly chosen 5% of reflections, which were not used for structure refinement, and R_work_ is calculated for the remaining reflections

^[f]^ Deviations from ideal bond lengths/angles

^[g]^ Percentage of residues in favored / allowed / outlier region

**Table S6|** Crystallographic data collection and refinement statistics.

|  | **CysG^ΔN16^-H122A:SAH** | **CysG^ΔN16^-H122N:SAH** | **CysG^ΔN16^-D190N:SAH** |
| --- | --- | --- | --- |
|  |  |  |  |
| **Crystal parameters** |  |  |  |
| Space group | P42_1_2 | P2_1_2_1_2 | P42_1_2 |
| Cell constants | a = 100.0 Å  b = 100.0 Å  c = 49.7 Å | a = 100.6 Å  b = 100.8 Å  c = 49.8 Å | a = 100.7 Å  b = 100.7 Å  c = 50.0 Å |
| molecules / AU^a^ | 1 | 2 | 1 |
|  |  |  |  |
| **Data collection** |  |  |  |
| Beam line | X06SA, SLS | X06SA, SLS | X06SA, SLS |
| Wavelength (Å) | 1.0 | 1.0 | 1.0 |
| Resolution range (Å)^b^ | 30-1.4 (1.5-1.4) | 30-1.6 (1.7-1.6) | 30-1.4 (1.5-1.4) |
| No. observations | 262809 | 293742 | 273604 |
| No. unique reflections^c^ | 50023 | 67193 | 50991 |
| Completeness (%)^b^ | 99.7 (100) | 99.2 (99.8) | 99.7 (99.9) |
| R_merge_ (%)^b, d^ | 6.5 (64.8) | 6.4 (65.0) | 4.8 (67.0) |
| I/σ (I)^b^ | 11.8 (2.3) | 12.1 (2.5) | 15.4 (2.5) |
|  |  |  |  |
| **Refinement (REFMAC5)** |  |  |  |
| Resolution range (Å) | 30-1.4 | 30-1.6 | 30-1.4 |
| No. refl. working set | 47483 | 63771 | 484014 |
| No. refl. test set | 2499 | 3357 | 2547 |
| No. non hydrogen | 1874 | 3673 | 1920 |
| No. of ligand atoms | 26 | 52 | 26 |
| No. of calcium ions | 1 (weak) | 0 | 1 |
| Solvent (H_2_O, ions, buffer) | 177 | 275 | 218 |
| R_work_/R_free_ (%)^e^ | 16.9/19.7 | 15.4/18.6 | 14.9/17.6 |
| r.m.s.d. bond (Å) / angle (°)^f^ | 0.004/1.3 | 0.004/1.2 | 0.004/1.2 |
| Average B-factor (Å^2^) | 19.8 | 22.6 | 21.3 |
| Ramachandran Plot (%)^g^ | 98.6/1.4/0 | 98.3/1.7/0 | 99.1/0.9/0 |
|  |  |  |  |
| PDB accession code | 9FCQ | 9FCS | 9FD3 |

^[a]^ Asymmetric unit

^[b]^ The values in parentheses for resolution range, completeness, R_merge_ and I/σ (I) correspond to the highest resolution shell

^[c]^ Data reduction was carried out from a single crystal. Friedel pairs were treated as identical reflections

^[d]^ R_merge_(I) = Σ_hkl_Σ_j_ | I(hkl)_j_ - <I(hkl)> | / Σ_hkl_ Σ_j_ I(hkl)_j_, where I(hkl)_j_ is the j^th^ measurement of the intensity of reflection hkl and <I(hkl)> is the average intensity

^[e]^ R = Σ_hkl_ | |F_obs_| - |F_calc_| |/Σ_hkl_ |F_obs_|, where R_free_ is calculated without a sigma cut off for a randomly chosen 5% of reflections, which were not used for structure refinement, and R_work_ is calculated for the remaining reflections

^[f]^ Deviations from ideal bond lengths/angles

^[g]^ Percentage of residues in favored / allowed / outlier region

**Table S7|** Crystallographic data collection and refinement statistics.

|  | **CysG^ΔN16^:SAH (metal-free)** |
| --- | --- |
|  |  |
| **Crystal parameters** |  |
| Space group | P2_1_2_1_2 |
| Cell constants | a = 99.6 Å  b = 103.1 Å  c = 50.0 Å |
| molecules / AU^a^ | 2 |
|  |  |
| **Data collection** |  |
| Beam line | P13, DESY |
| Wavelength (Å) | 0.976 |
| Resolution range (Å)^b^ | 30-2.0 (2.1-2.0) |
| No. observations | 121128 |
| No. unique reflections^c^ | 34551 |
| Completeness (%)^b^ | 97.1 (98.6) |
| R_merge_ (%)^b, d^ | 9.6 (59.6) |
| I/σ (I)^b^ | 8.0 (2.1) |
|  |  |
| **Refinement (REFMAC5)** |  |
| Resolution range (Å) | 30-2.0 |
| No. refl. working set | 32778 |
| No. refl. test set | 1726 |
| No. non hydrogen | 3427 |
| No. of ligand atoms | 52 |
| No. of calcium ions | 0 |
| Solvent (H_2_O, ions, buffer) | 23 |
| R_work_/R_free_ (%)^e^ | 21.4/26.1 |
| r.m.s.d. bond (Å) / angle (°)^f^ | 0.014/1.8 |
| Average B-factor (Å^2^) | 35.7 |
| Ramachandran Plot (%)^g^ | 97.4/2.6/0 |
|  |  |
| PDB accession code | 9G0K |

^[a]^ Asymmetric unit

^[b]^ The values in parentheses for resolution range, completeness, R_merge_ and I/σ (I) correspond to the highest resolution shell

^[c]^ Data reduction was carried out from a single crystal. Friedel pairs were treated as identical reflections

^[d]^ R_merge_(I) = Σ_hkl_Σ_j_ | I(hkl)_j_ - <I(hkl)> | / Σ_hkl_ Σ_j_ I(hkl)_j_, where I(hkl)_j_ is the j^th^ measurement of the intensity of reflection hkl and <I(hkl)> is the average intensity

^[e]^ R = Σ_hkl_ | |F_obs_| - |F_calc_| |/Σ_hkl_ |F_obs_|, where R_free_ is calculated without a sigma cut off for a randomly chosen 5% of reflections, which were not used for structure refinement, and R_work_ is calculated for the remaining reflections

^[f]^ Deviations from ideal bond lengths/angles

^[g]^ Percentage of residues in favored / allowed / outlier region


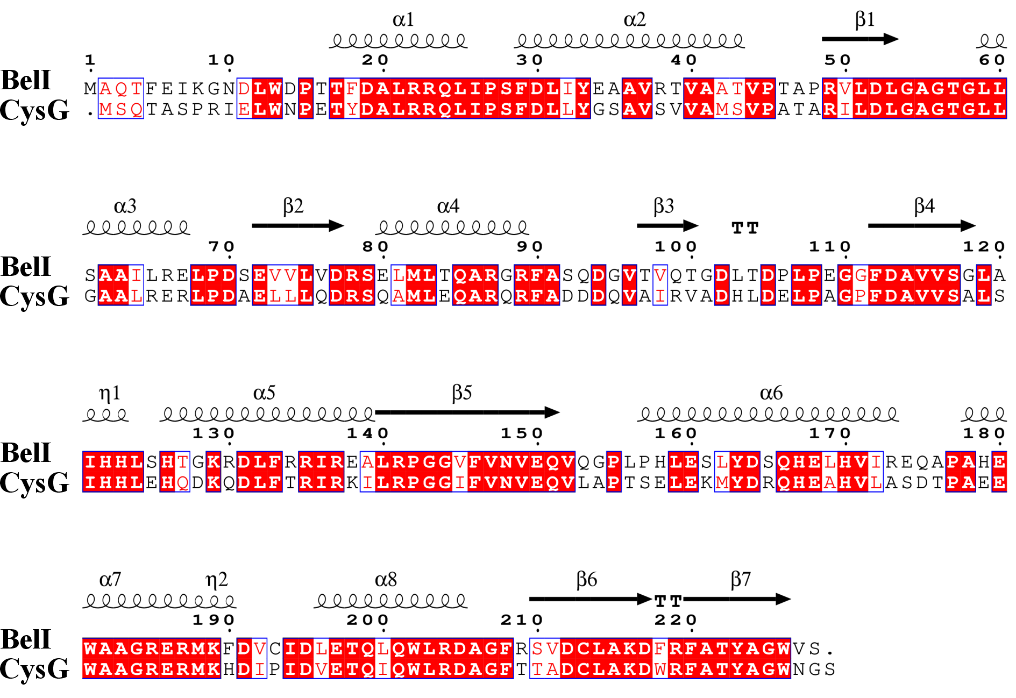


**Figure S1| Primary sequence alignment of methyltransferases BelI and CysG.** The protein sequences of BelI and CysG share 66% identity according to ESPript3.0 *(1)*. Secondary structure elements are indicated according to the structure of BelI. For clarity reasons, enumeration was referred to the primary sequence of BelI.


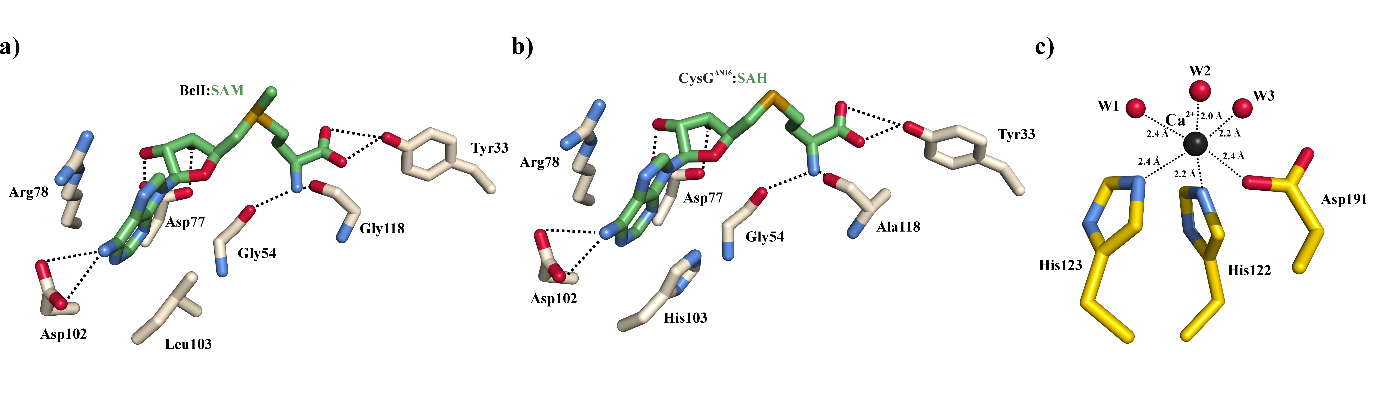


**Figure S2| Structural analysis of cosubstrate and metal coordination.** Representation of protein interactions of **a)** SAM bound to BelI (PDB ID: 9FCE) and **b)** SAH bound to CysG^ΔN16^ (PDB ID: 9FCD)(carbon atoms of SAM/SAH in green and proteins residues in grey; oxygen in red; nitrogen in blue; sulfur in yellow). Dots illustrate hydrogen bonds of the cosubstrates with protein residues. Arg78 further stabilizes the adenine portion by cation-π-interactions. **c)** Illustration of the octahedral coordinated divalent metal binding site in CysG^ΔN16^ (PDB ID: 9FCD) at 1.5 Å (calcium ion in black, interacting carbon atoms in gold, and water molecules in red). Geometric analysis reveals bond lengths and angles typical for calcium *(2)*.


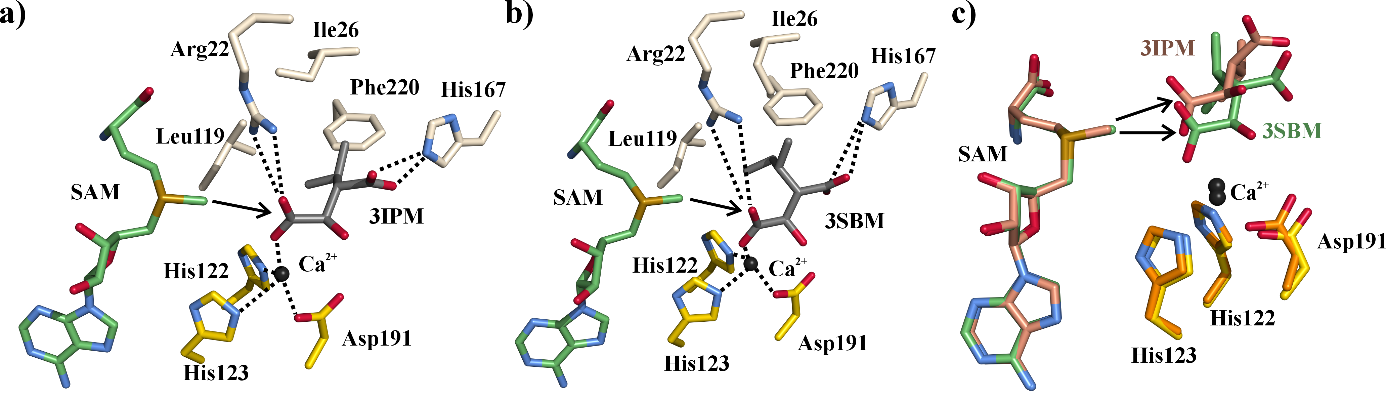


**Figure S3| Binding mode predictions of 3IPM and 3SBM within the active sites of CysG and BelI.** Cartoon illustration of the binding mode predicted with Autodock Vina *(3,4)* of **a)** CysG in complex with 3IPM and **b)** BelI with 3SBM and SAM. The C1-carboxy group of the substrates interacts with Arg22 and the calcium ion (Ca^2+^, black). The C4-carboxy group forms hydrogen bonds with His167 and the aliphatic side chain of 3IPM/3SBM is stabilized within an apolar pocket formed by Ile26, Leu119, and Phe220. Dots represent interactions between the substrates and the metal ion, cosubstrate, as well as protein residues. Arrows highlight the methyl transfer trajectory. **c)** Superposition of modeled 3IPM (salmon) and 3SBM (green) reveal identical interactions within the active sites of CysG and BelI.


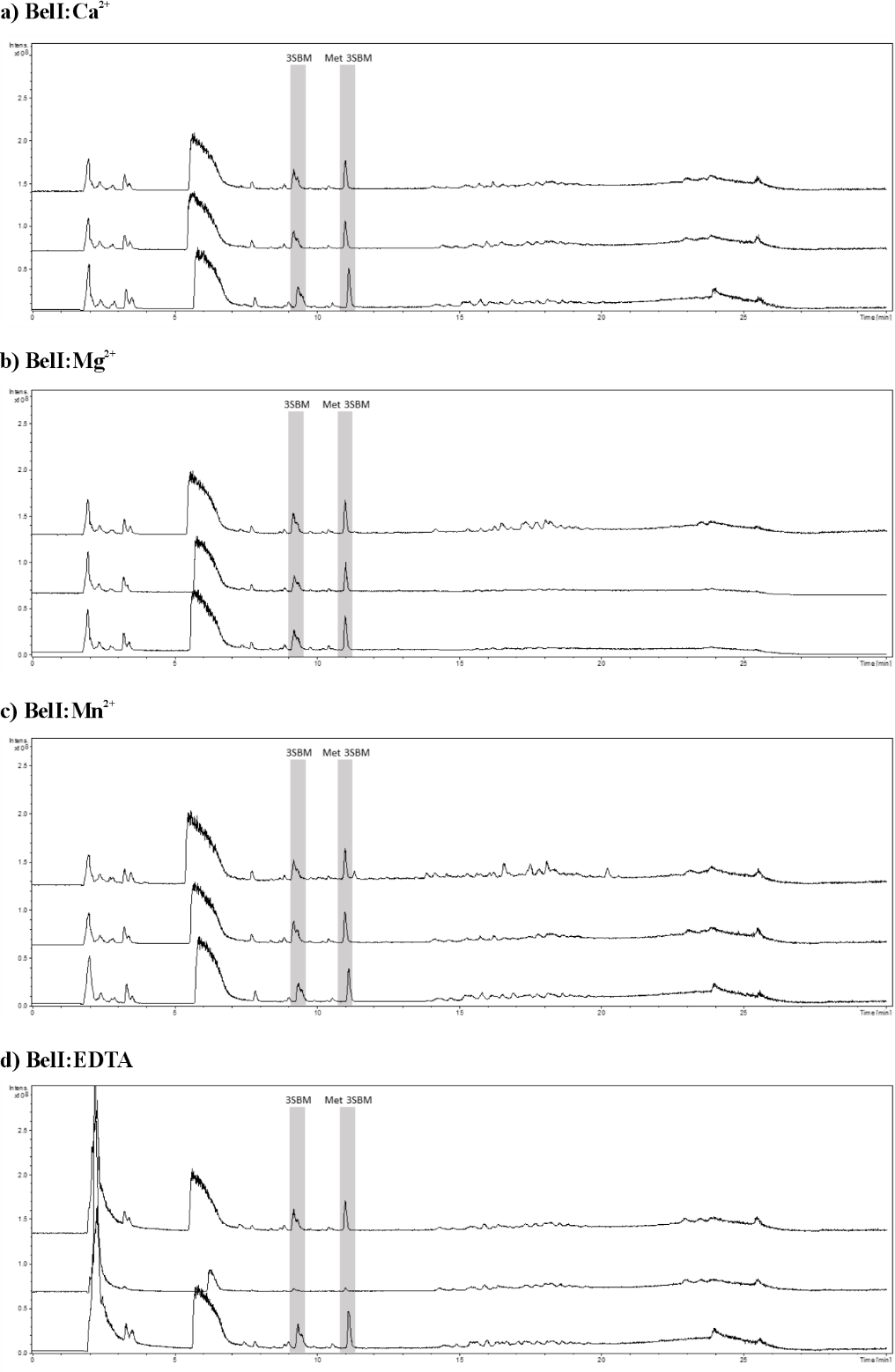


**Figure S4| LC/MS analysis of in vitro 3-SBM methylation by BelI.** The reaction was performed in the presence of SAM as cofactor and a) CaCl_2_, b) MgCl_2_, c) MnCl_2_, or d) EDTA in the reaction buffer (n=3). Displayed are the total ion chromatograms (TICs), highlighted in gray are the substrate 3-secbutylmalate and the product methylated 3-secbutylmalate.


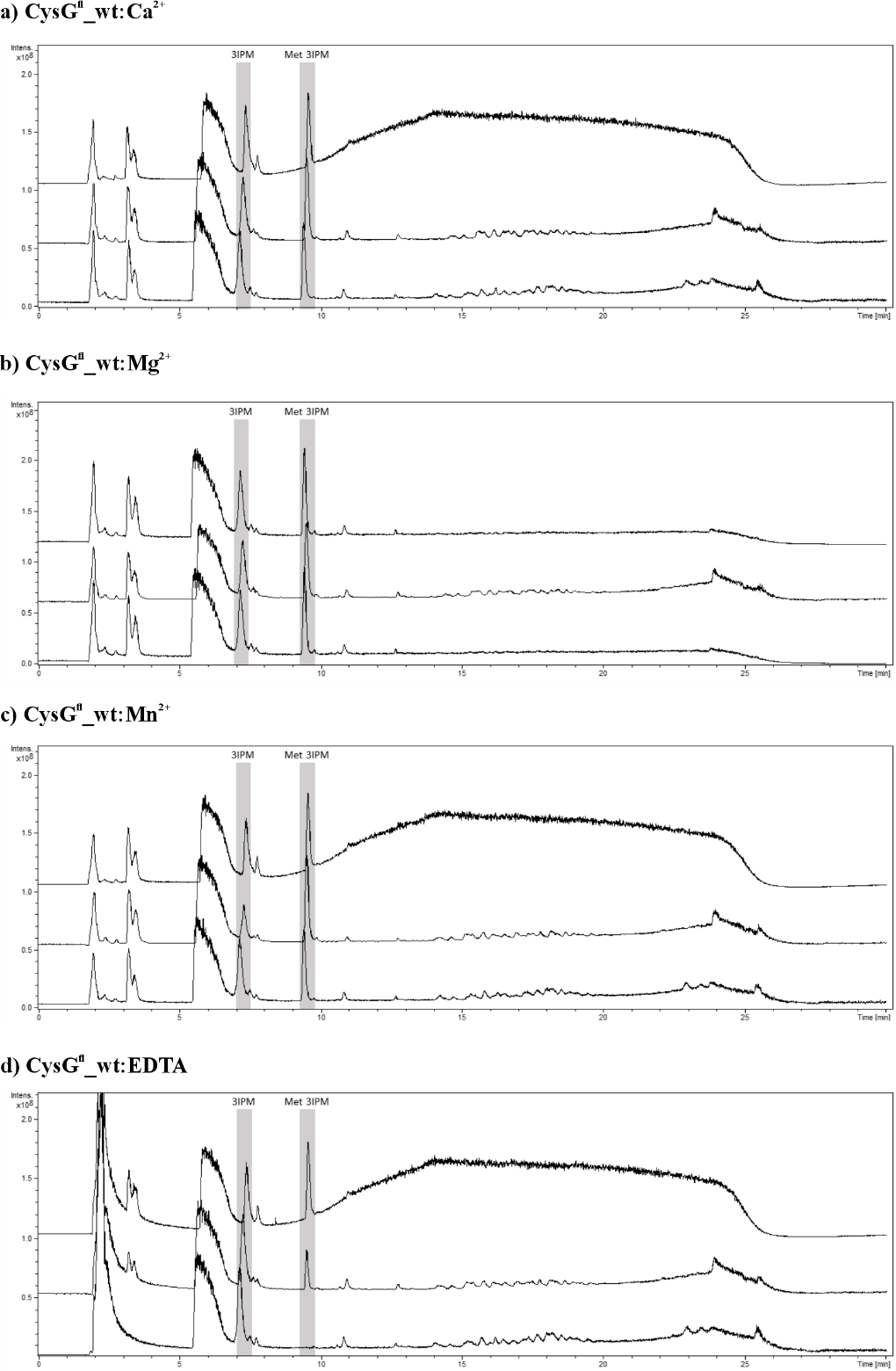


**Figure S5| LC/MS analysis of in vitro 3-IPM methylation by CysG^fl^ wildtype.** The reaction was performed in the presence of SAM as cofactor and a) CaCl_2_, b) MgCl_2_, c) MnCl_2_, or d) EDTA in the reaction buffer (n=3). Displayed are the total ion chromatograms (TICs), highlighted in gray are the substrate 3-isopropylmalate and the product methylated 3-isopropylmalate.

**
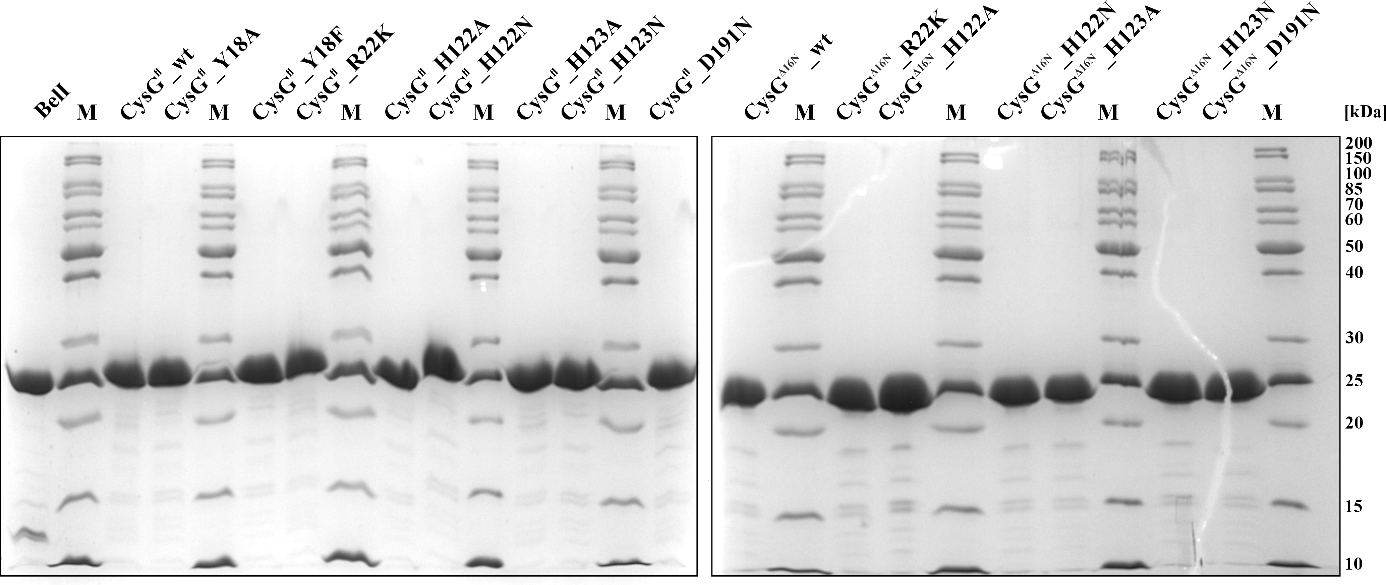
**

**Figure S6| SDS-gel analysis of purified enzymes.** Coomassie stained SDS-gel (15%) of BelI, CysG, CysG^ΔN16^, and mutant variants. 5 ng of purified protein were analyzed on two SDS-gels marked by black rectangles.


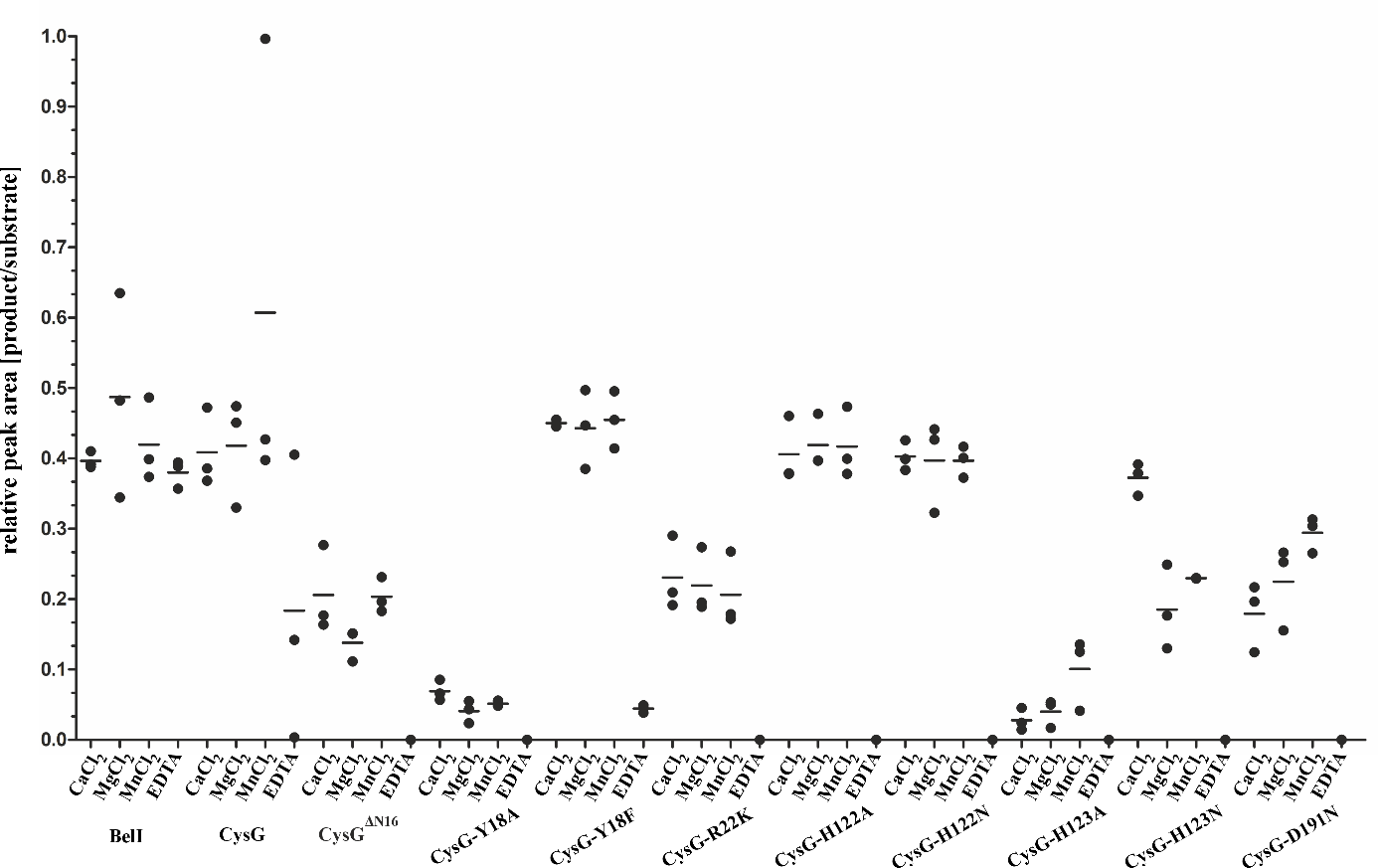


**Figure S7: Enzymatic Reactions with Divalent Metal Ions.** Representation of the relative peak areas in the total ion chromatograms (TICs) of the products and substrate in three independent HPLC-MS measurements and their mean values, conducted with BelI, CysG^fl^, CysG^ΔN16^ and CysG variants, either showing conversion of 3IPM to 1-methyl-3IPM or 3SBM to 1-methyl-3-SBM.

**
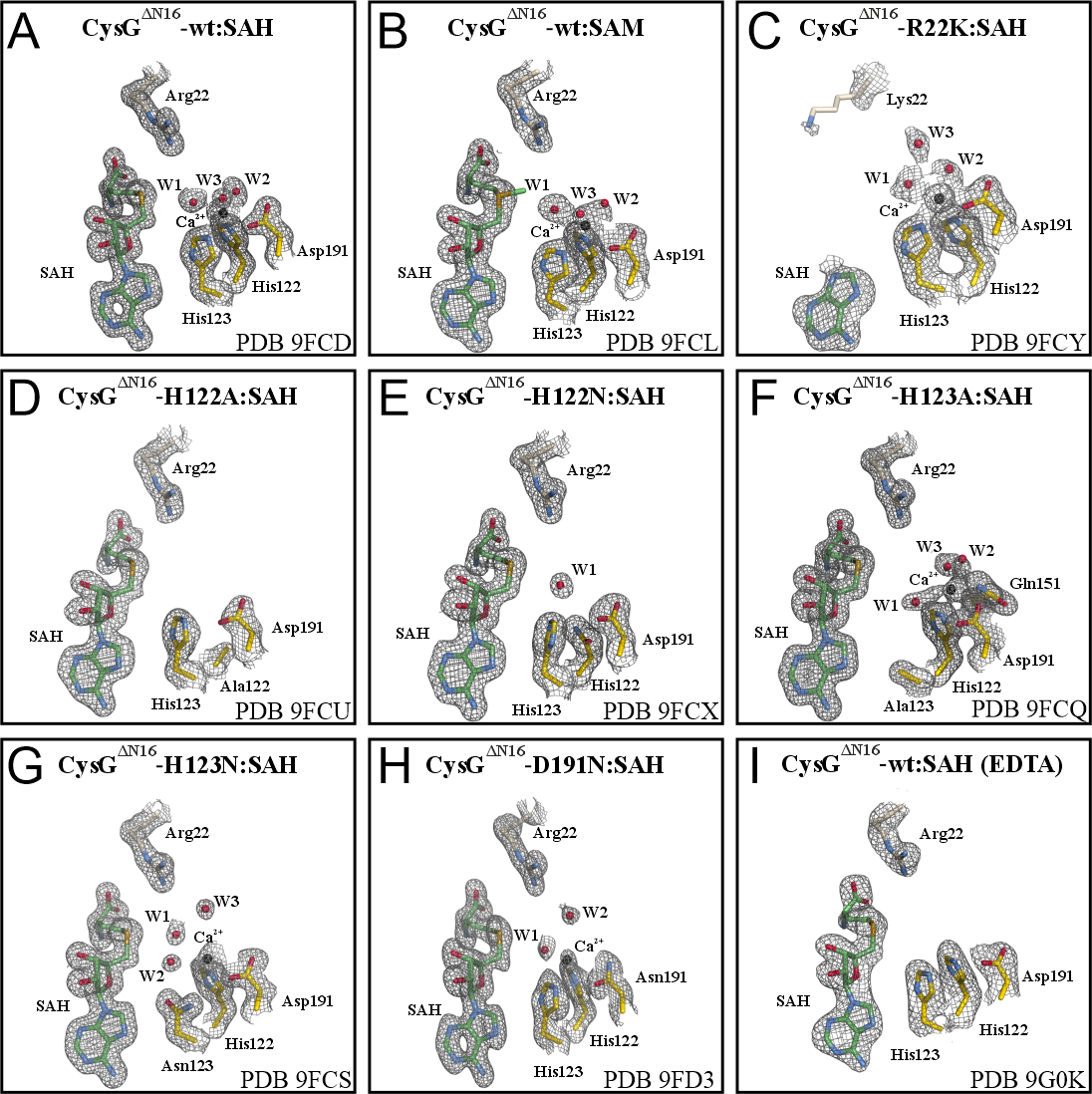
**

**Figure S8| Structural analysis of the active site of CysG^ΔN16^ and its mutants. (A-I)** 2F_O_-F_C_ electron densities (gray meshes, contoured to 1σ) of CysG^ΔN16^ and its mutants in complex with the enzyme-bound cofactor SAH/SAM (carbon atoms in green, oxygen in red, nitrogen in blue, sulfur in yellow), the calcium ion (black), and its metal binding site (carbon atoms in gold) are depicted. W1-W3 denote water molecules.

**
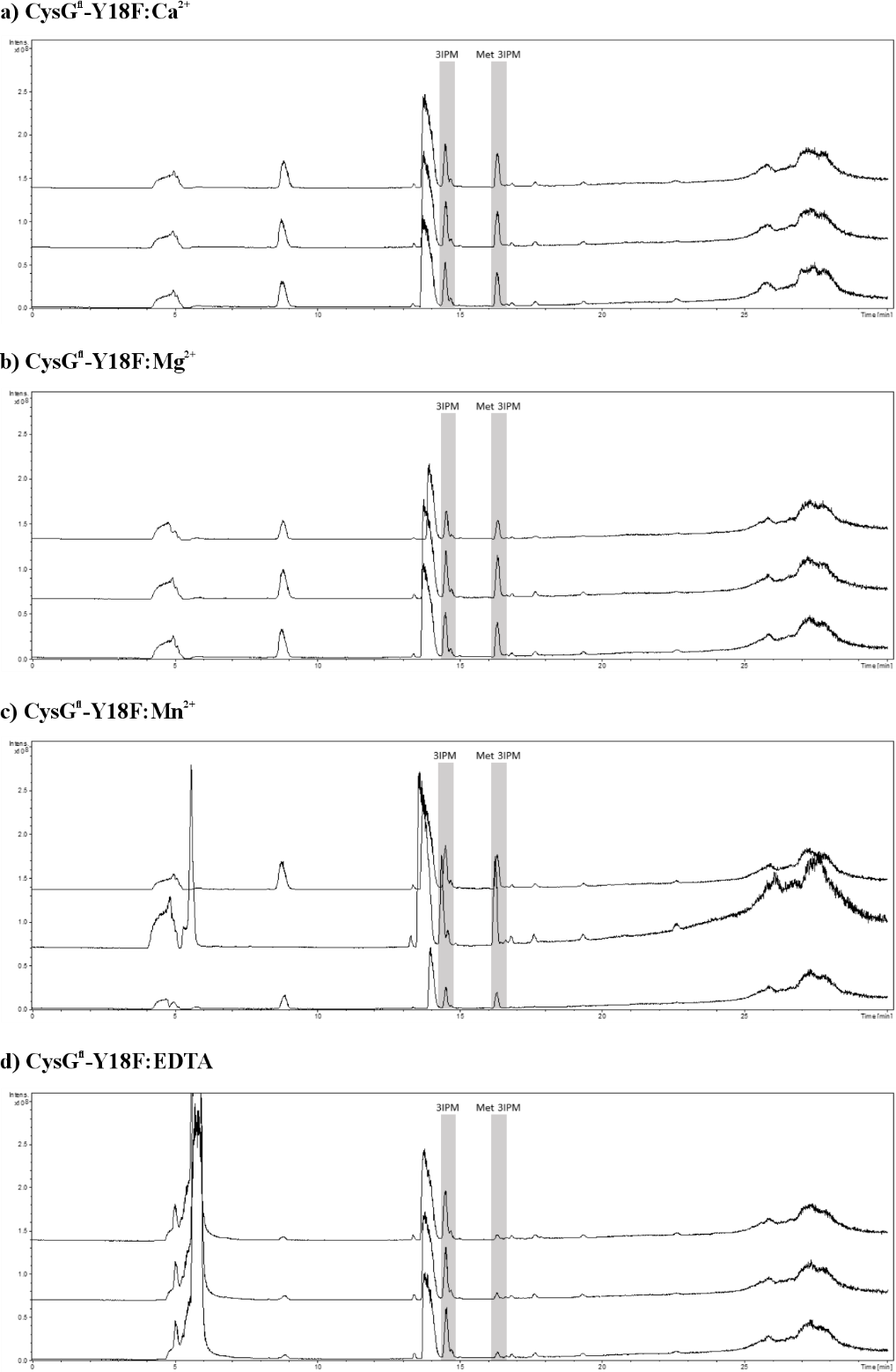
**

**Figure S9| LC/MS analysis of in vitro 3-IPM methylation by CysG^fl^-Y18F.** The reaction was performed in the presence of SAM as cofactor and a) CaCl_2_, b) MgCl_2_, c) MnCl_2_, or d) EDTA in the reaction buffer (n=3). Displayed are the total ion chromatograms (TICs), highlighted in gray are the substrate 3-isopropylmalate and the product methylated 3-isopropylmalate.


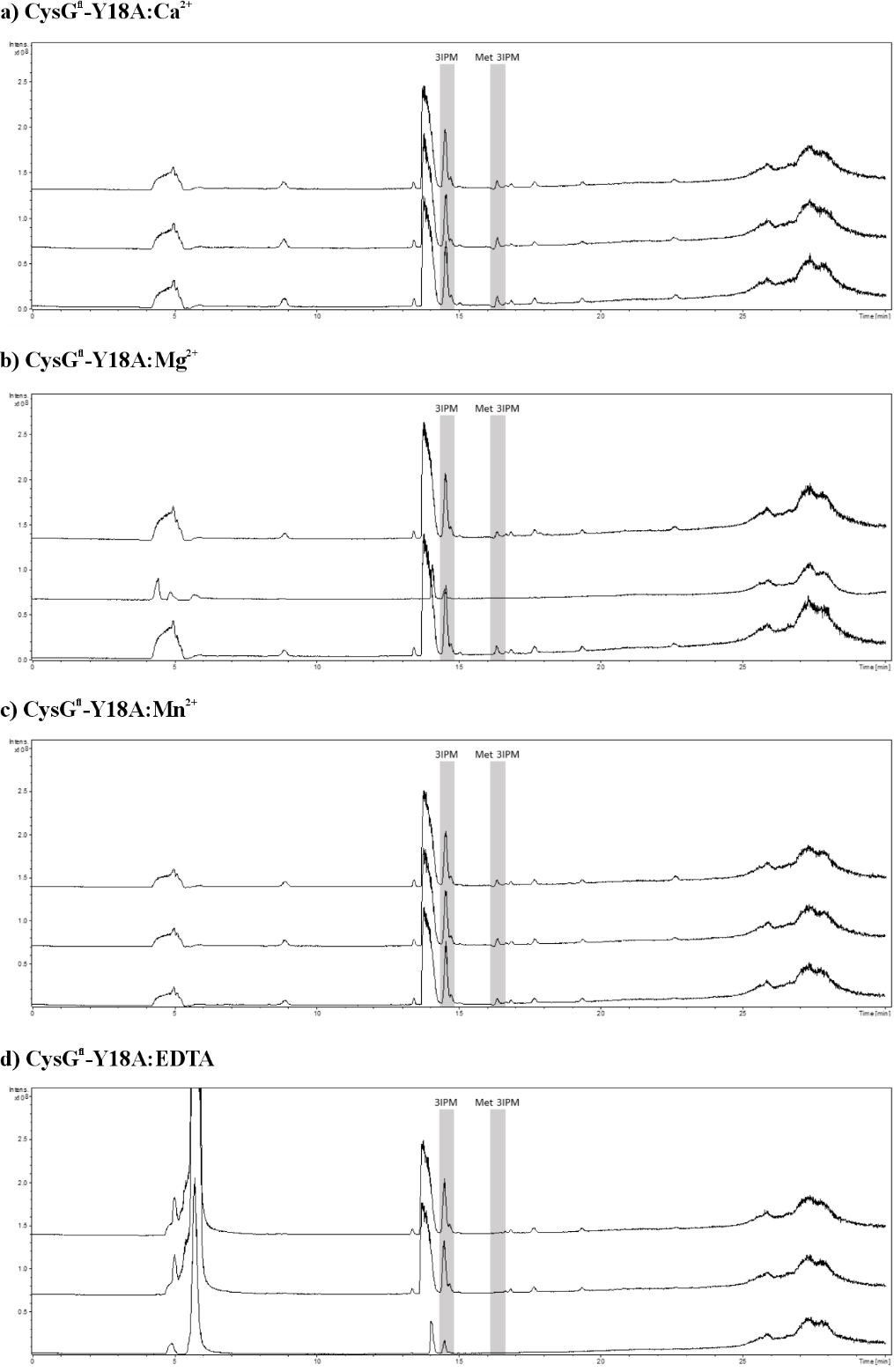


**Figure S10| LC/MS analysis of in vitro 3-IPM methylation by CysG^fl^-Y18A.** The reaction was performed in the presence of SAM as cofactor and a) CaCl_2_, b) MgCl_2_, c) MnCl_2_, or d) EDTA in the reaction buffer (n=3). Displayed are the total ion chromatograms (TICs), highlighted in gray are the substrate 3-isopropylmalate and the product methylated 3-isopropylmalate.


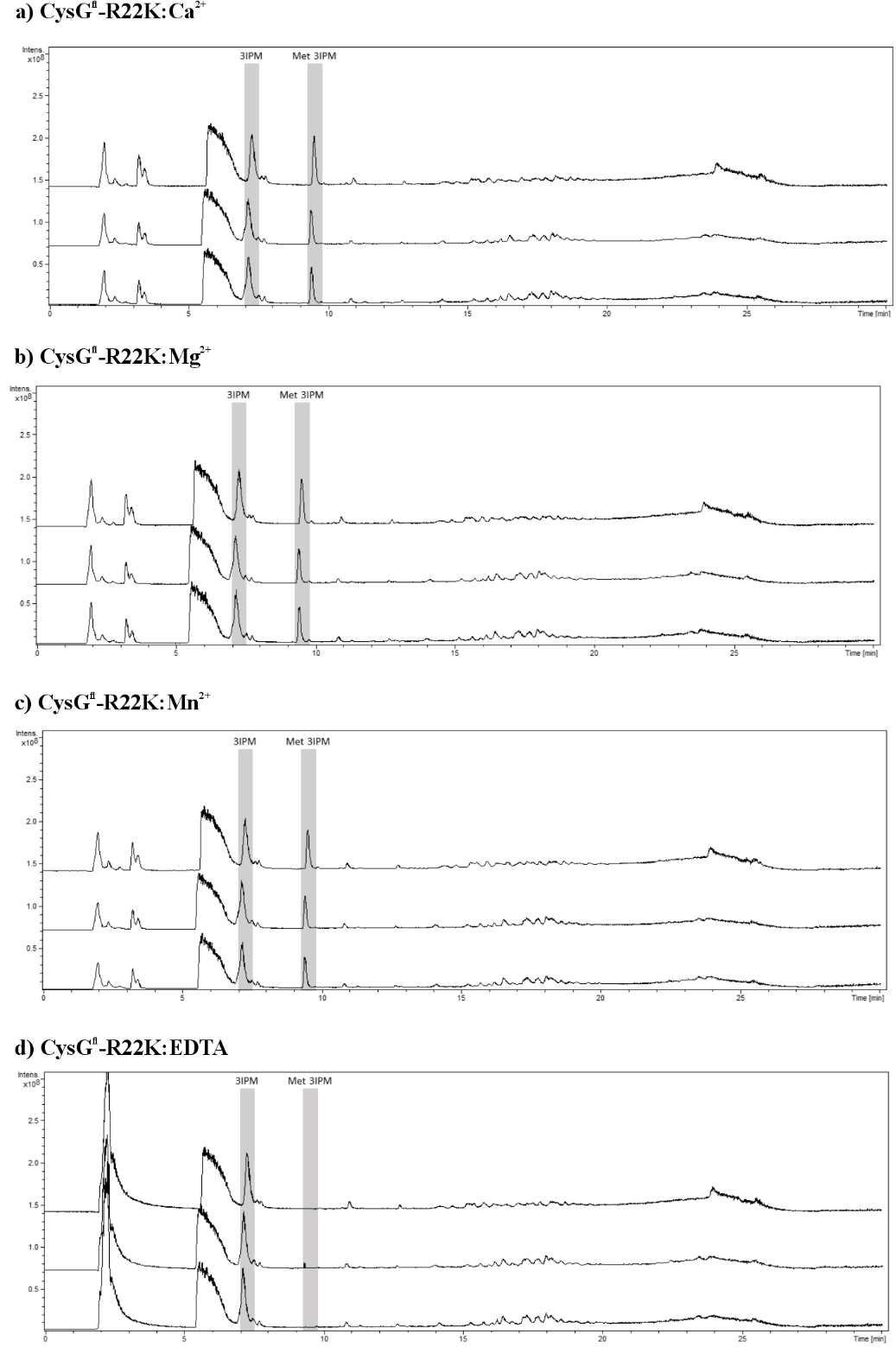


**Figure S11| LC/MS analysis of in vitro 3-IPM methylation by CysG^fl^-R22K.** The reaction was performed in the presence of SAM as cofactor and a) CaCl_2_, b) MgCl_2_, c) MnCl_2_, or d) EDTA in the reaction buffer (n=3). Displayed are the total ion chromatograms (TICs), highlighted in gray are the substrate 3-isopropylmalate and the product methylated 3-isopropylmalate.


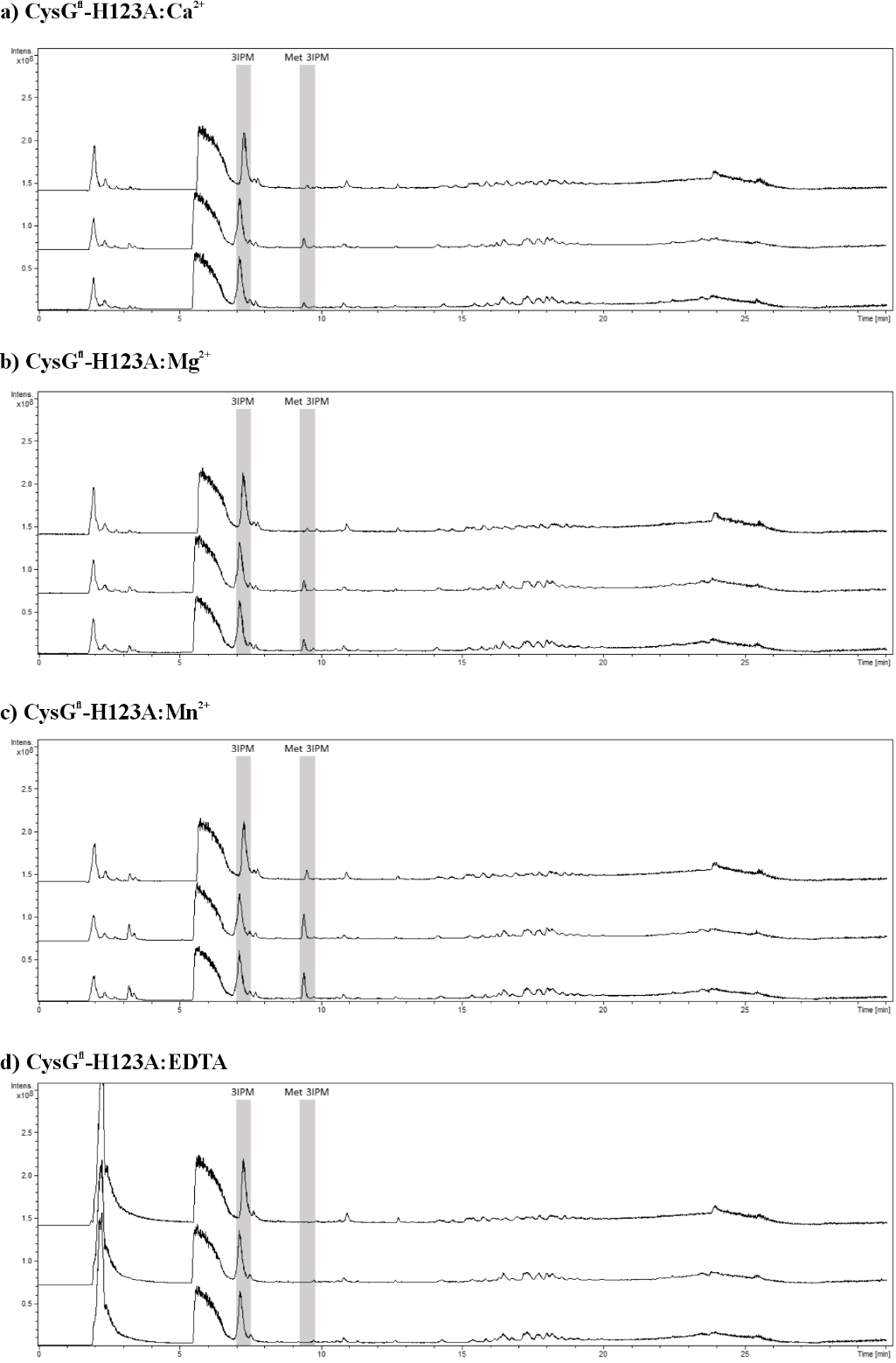


**Figure S12| LC/MS analysis of in vitro 3-IPM methylation by CysG^fl^-H123A.** The reaction was performed in the presence of SAM as cofactor and a) CaCl_2_, b) MgCl_2_, c) MnCl_2_, or d) EDTA in the reaction buffer (n=3). Displayed are the total ion chromatograms (TICs), highlighted in gray are the substrate 3-isopropylmalate and the product methylated 3-isopropylmalate.


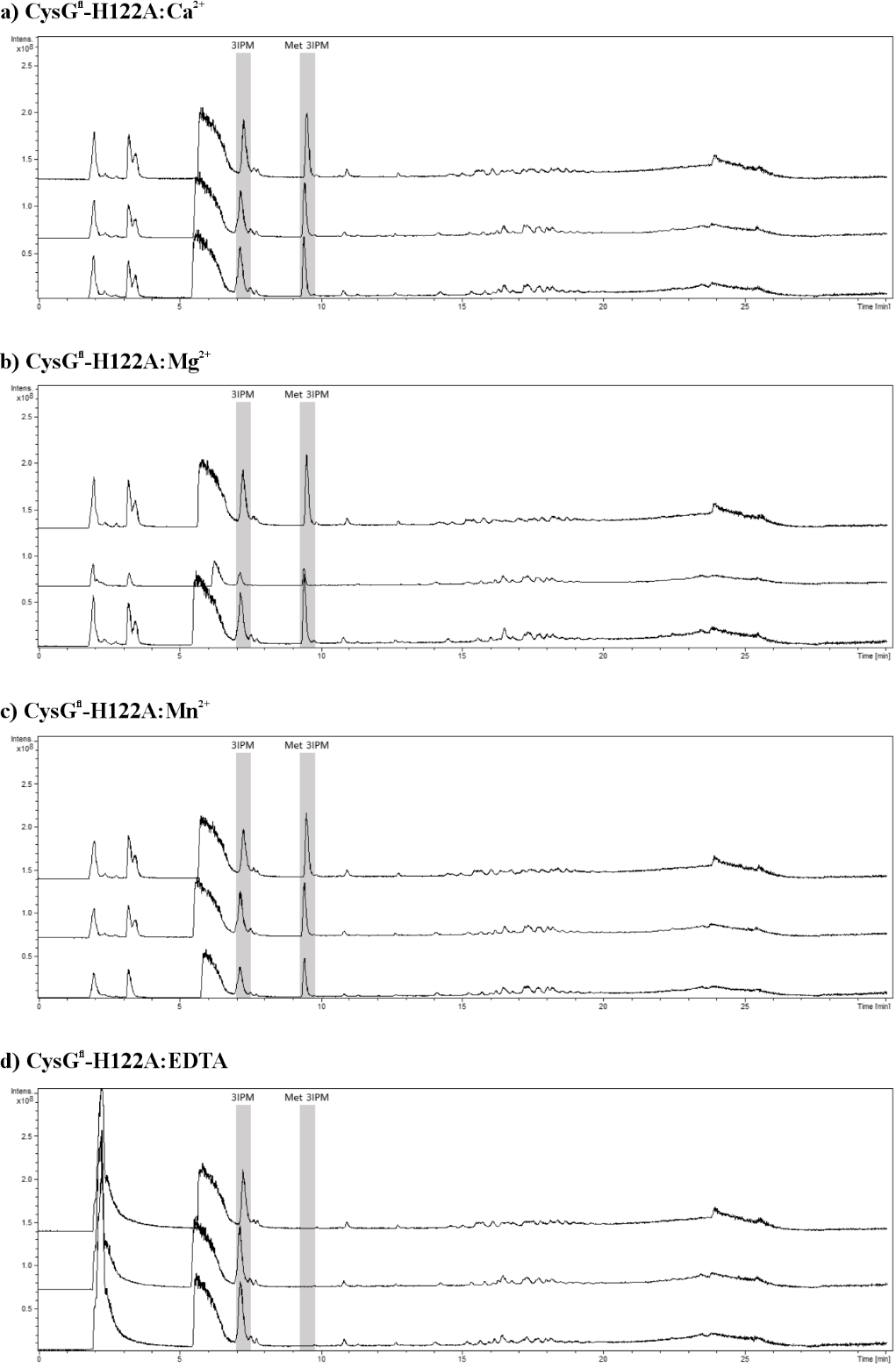


**Figure S13| LC/MS analysis of in vitro 3-IPM methylation by CysG^fl^-H122A.** The reaction was performed in the presence of SAM as cofactor and a) CaCl_2_, b) MgCl_2_, c) MnCl_2_, or d) EDTA in the reaction buffer (n=3). Displayed are the total ion chromatograms (TICs), highlighted in gray are the substrate 3-isopropylmalate and the product methylated 3-isopropylmalate.


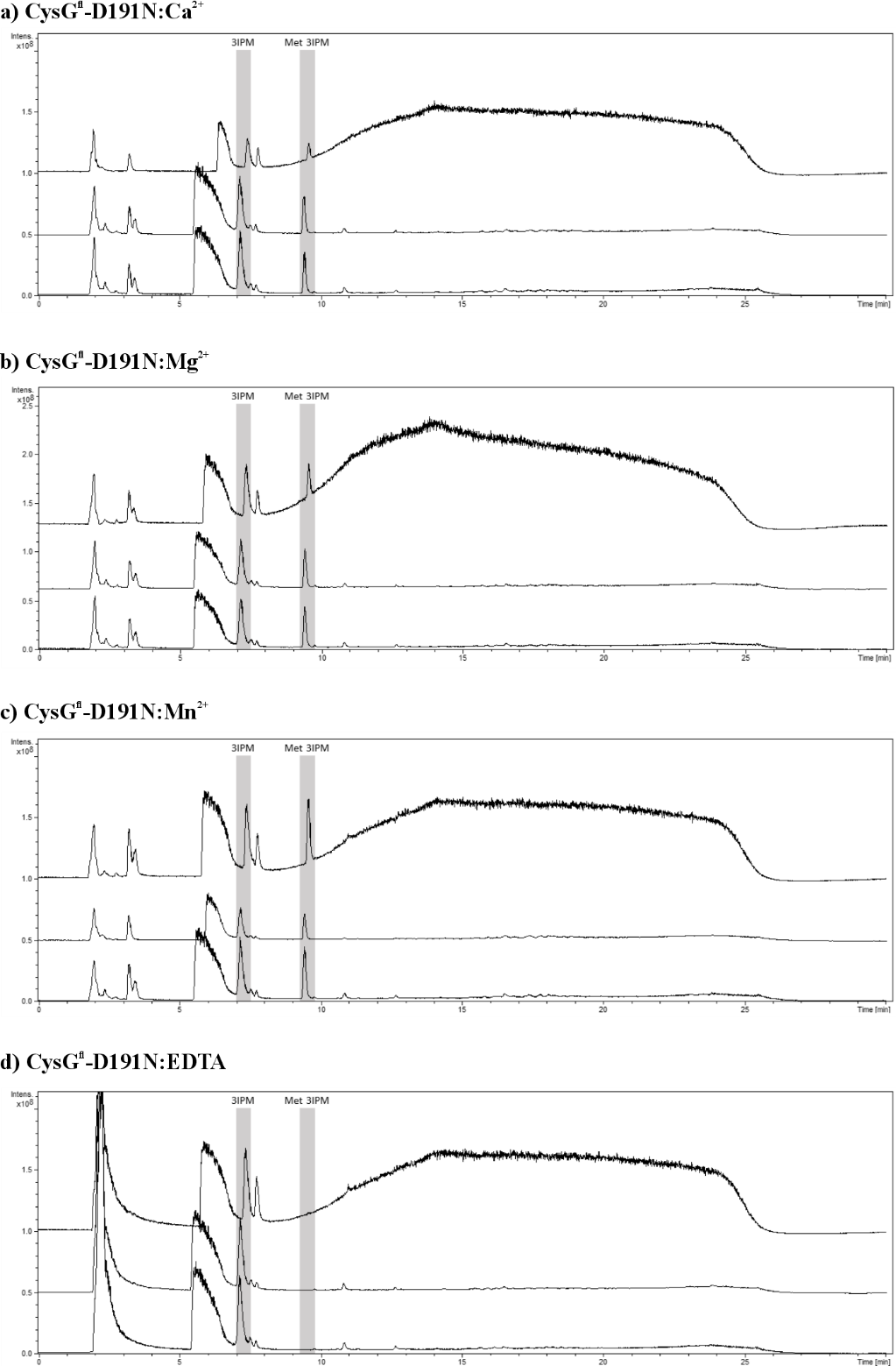


**Figure S14| LC/MS analysis of in vitro 3-IPM methylation by CysG^fl^-D191N.** The reaction was performed in the presence of SAM as cofactor and a) CaCl_2_, b) MgCl_2_, c) MnCl_2_, or d) EDTA in the reaction buffer (n=3). Displayed are the total ion chromatograms (TICs), highlighted in gray are the substrate 3-isopropylmalate and the product methylated 3-isopropylmalate.


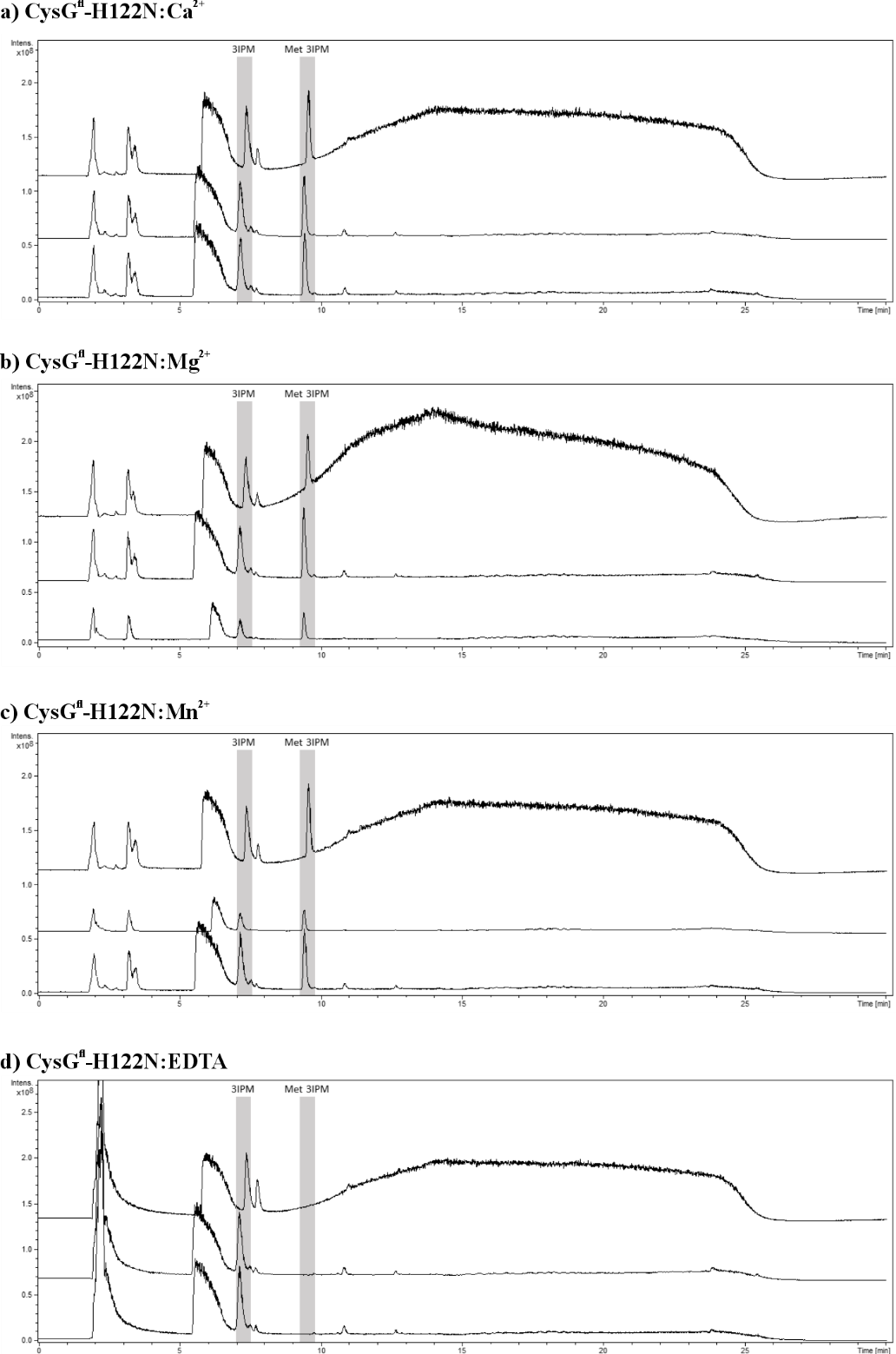


**Figure S15| LC/MS analysis of in vitro 3-IPM methylation by CysG^fl^-H122N.** The reaction was performed in the presence of SAM as cofactor and a) CaCl_2_, b) MgCl_2_, c) MnCl_2_, or d) EDTA in the reaction buffer (n=3). Displayed are the total ion chromatograms (TICs), highlighted in gray are the substrate 3-isopropylmalate and the product methylated 3-isopropylmalate.

**
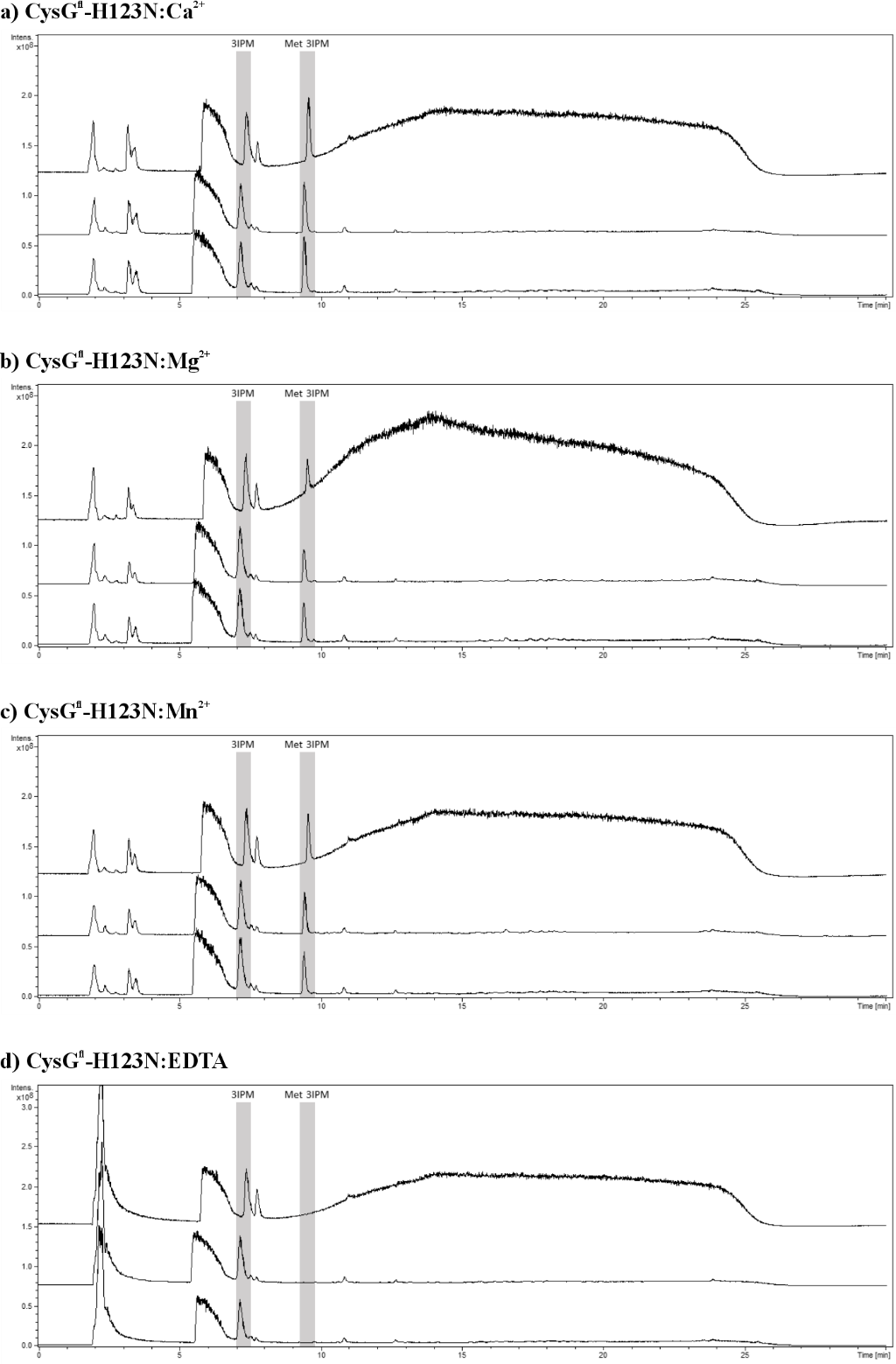
**

**Figure S16| LC/MS analysis of in vitro 3-IPM methylation by CysG^fl^-H123N.** The reaction was performed in the presence of SAM as cofactor and a) CaCl_2_, b) MgCl_2_, c) MnCl_2_, or d) EDTA in the reaction buffer (n=3). Displayed are the total ion chromatograms (TICs), highlighted in gray are the substrate 3-isopropylmalate and the product methylated 3-isopropylmalate.

**
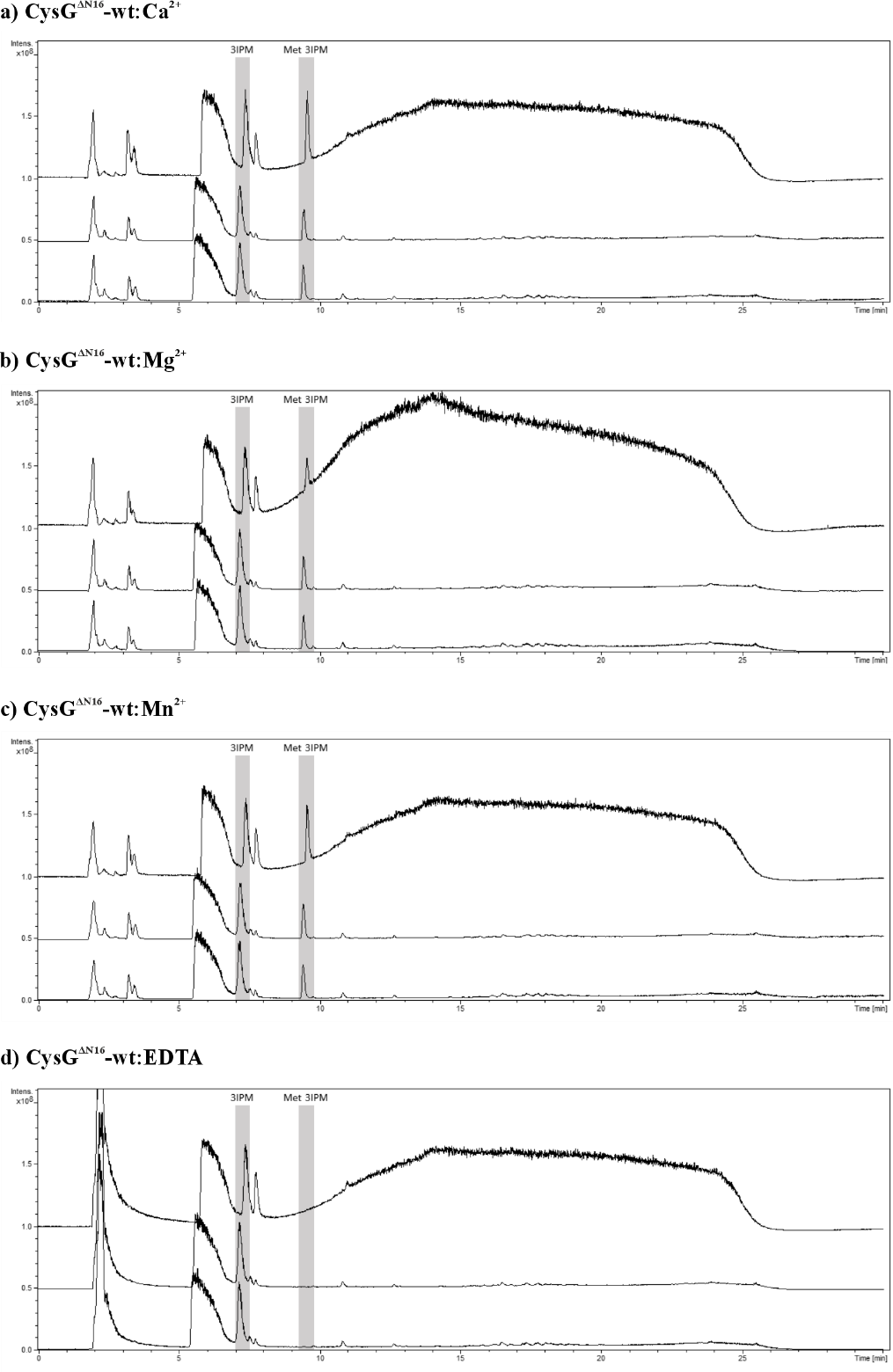
**

**Figure S17| LC/MS analysis of in vitro 3-IPM methylation by CysG^ΔN16^ wildtype.** The reaction was performed in the presence of SAM as cofactor and a) CaCl_2_, b) MgCl_2_, c) MnCl_2_, or d) EDTA in the reaction buffer (n=3). Displayed are the total ion chromatograms (TICs), highlighted in gray are the substrate 3-isopropylmalate and the product methylated 3-isopropylmalate.


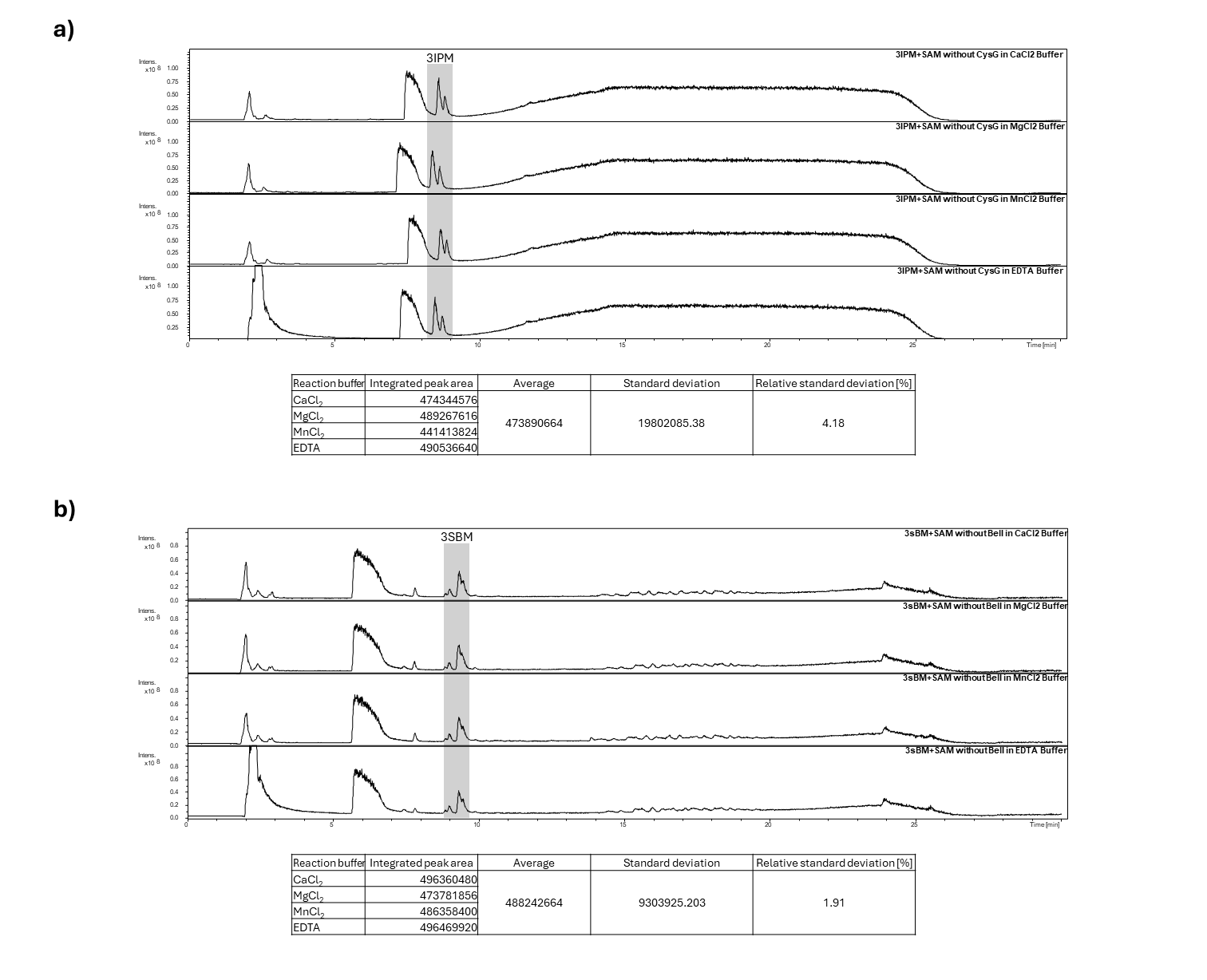


**Figure S18| LC/MS analysis of enzyme-free assay controls.** The reactions were performed with a) 3IPM and b) 3SBM in the presence of SAM as cofactor and CaCl_2_, MgCl_2_, MnCl_2_, or EDTA in the reaction buffer. Displayed are the total ion chromatograms (TICs), highlighted in gray are the substrates. Low standard deviations of the integrated peak area average apply to both 3IPM and 3SBM and support the conclusion that variation of extraction efficiencies is minor.

References

1. Robert, X., and Gouet, P. (2014) Deciphering key features in protein structures with the new ENDscript server, *Nucleic acids research 42,* W320-4.

2. Cates, M. S., Teodoro, M. L., and Phillips, G. N. (2002) Molecular mechanisms of calcium and magnesium binding to parvalbumin, *Biophysical journal 82,* 1133–1146.

3. Eberhardt, J., Santos-Martins, D., Tillack, A. F., and Forli, S. (2021) AutoDock Vina 1.2.0: New Docking Methods, Expanded Force Field, and Python Bindings, *Journal of chemical information and modeling 61,* 3891–3898.

4. Trott, O., and Olson, A. J. (2010) AutoDock Vina: improving the speed and accuracy of docking with a new scoring function, efficient optimization, and multithreading, *Journal of computational chemistry 31,* 455–461.
